# Ferret models of alpha-1 antitrypsin deficiency develop lung and liver disease

**DOI:** 10.1172/jci.insight.143004

**Published:** 2022-03-08

**Authors:** Nan He, Xiaoming Liu, Amber R. Vegter, T. Idil A. Evans, Jaimie S. Gray, Junfeng Guo, Shashanna R. Moll, Lydia J. Guo, Meihui Luo, Ningxia Ma, Xingshen Sun, Bo Liang, Ziying Yan, Zehua Feng, Lisi Qi, Arnav S. Joshi, Weam Shahin, Yaling Yi, Katherine N. Gibson-Corley, Eric A. Hoffman, Kai Wang, Christian Mueller, John F. Engelhardt, Bradley H. Rosen

**Affiliations:** 1 Department of Anatomy and Cell Biology,; 2 Department of Radiology,; 3 Department of Surgery,; 4 Department of Pathology, and; 5 Department of Biostatistics, University of Iowa Carver College of Medicine, Iowa City, Iowa, USA.; 6 Department of Pediatrics, University of Massachusetts Medical Center, Worcester, Massachusetts, USA.; 7 Department of Internal Medicine, University of Iowa Carver College of Medicine, Iowa City, Iowa, USA.

**Keywords:** Pulmonology, COPD, Genetic diseases, Proteases

## Abstract

Alpha-1 antitrypsin deficiency (AATD) is the most common genetic cause and risk factor for chronic obstructive pulmonary disease, but the field lacks a large-animal model that allows for longitudinal assessment of pulmonary function. We hypothesized that ferrets would model human AATD-related lung and hepatic disease. AAT-knockout (AAT-KO) and PiZZ (E342K, the most common mutation in humans) ferrets were generated and compared with matched controls using custom-designed flexiVent modules to perform pulmonary function tests, quantitative computed tomography (QCT), bronchoalveolar lavage (BAL) proteomics, and alveolar morphometry. Complete loss of AAT (AAT-KO) led to increased pulmonary compliance and expiratory airflow limitation, consistent with obstructive lung disease. QCT and morphometry confirmed emphysema and airspace enlargement, respectively. Pathway analysis of BAL proteomics data revealed inflammatory lung disease and impaired cellular migration. The PiZ mutation resulted in altered AAT protein folding in the liver, hepatic injury, and reduced plasma concentrations of AAT, and PiZZ ferrets developed obstructive lung disease. In summary, AAT-KO and PiZZ ferrets model the progressive obstructive pulmonary disease seen in AAT-deficient patients and may serve as a platform for preclinical testing of therapeutics including gene therapy.

## Introduction

Alpha-1 antitrypsin deficiency (AATD) is the most common genetic risk factor for developing emphysema, a form of chronic obstructive pulmonary disease (COPD). COPD is the third leading cause of death worldwide (1, 2). AATD is caused by mutations in the SERine Proteinase INhibitor family A member 1 (*SERPINA1*) gene, which result in low circulating levels of AAT, a protease inhibitor that protects tissues from excessive protease activity (3–5). The bulk of AAT is produced by hepatocytes; additional sources that are expressed within the lung come from hematopoietic cells, epithelial cells in the distal airways, and type 2 alveolar cells (6–8). AAT is the second most abundant protein in blood plasma, and it increases in response to inflammation and physiological stress (4). In the lung, AAT counterbalances the activities of proteases such as neutrophil elastase (NE), cathepsin G, proteinase-3, and matrix metalloproteinases, which contribute to destruction of the distal airways and alveolar walls (9, 10). Although extraordinarily rare, *SERPINA1* mutations that completely abolish AAT protein expression have been identified (11, 12), and this absence of AAT leads to pulmonary emphysema and chronic bronchitis without liver disease (10, 13). AATD is more commonly caused by a point mutation (Z-allele) that leads to protein aggregation within hepatocytes, hepatotoxicity, and reduced AAT plasma levels (14–16). The Z-allele is distinguished from the normal AAT protein, called PiM, by its migration pattern under isoelectric focusing where the normal M-allele migrates faster than the mutant Z-allele. Humans homozygous for the Z-allele (PiZZ) are highly susceptible to excessive protease-mediated damage related to environmental exposures and inflammatory stimuli to the lung, particularly exposure to tobacco smoke (5).

The generation of a mouse model for AATD has been hampered by the existence of multiple *SERPINA1* paralogs in the murine genome (17–19). This hurdle was overcome by using CRISPR/Cas9 to generate a *SerpinA1a-e*–knockout mouse, and this model was found to develop emphysema spontaneously (20). However, mouse models of lung disease have 2 major limitations: (a) significant differences in the anatomy and cell biology with the human lung and (b) the short life span and small size of mice that make longitudinal analysis of pulmonary function challenging, although findings from microCT and other imaging techniques have been used as surrogate endpoints for disease progression (21, 22). Previously reported ferret models have a promising track record of advancing respiratory research, having shed light on the pathobiology of chronic bronchitis (23), cystic fibrosis (24–26), and chronic rejection of lung transplants (27). Given that large-animal models are most effective in longitudinal studies of disease, we hypothesized that AAT-knockout (AAT-KO) ferrets would allow for studies of both the etiology and progression of chronic pulmonary disease and that a Z-allele knockin (PiZZ) ferret could model both lung and hepatic aspects of the human disease.

As expected, AAT-KO ferrets developed spontaneous pulmonary emphysema and evidence of inflammation in the bronchoalveolar lavage (BAL) as assessed by proteomics. Given the ability of this ferret model to recapitulate the lung phenotype associated with AATD, we then created the PiZZ ferret to model the most common human *SERPINA1* mutation. Notably, we observed juvenile hepatotoxicity associated with Z-allele accumulation in hepatocytes as well as pulmonary disease, 2 phenotypes that are frequently associated with human AATD patients with the Z-allele mutations.

## Results

### Generation of SERPINA1-KO, or AAT-KO, ferrets.

**Figure 1:**
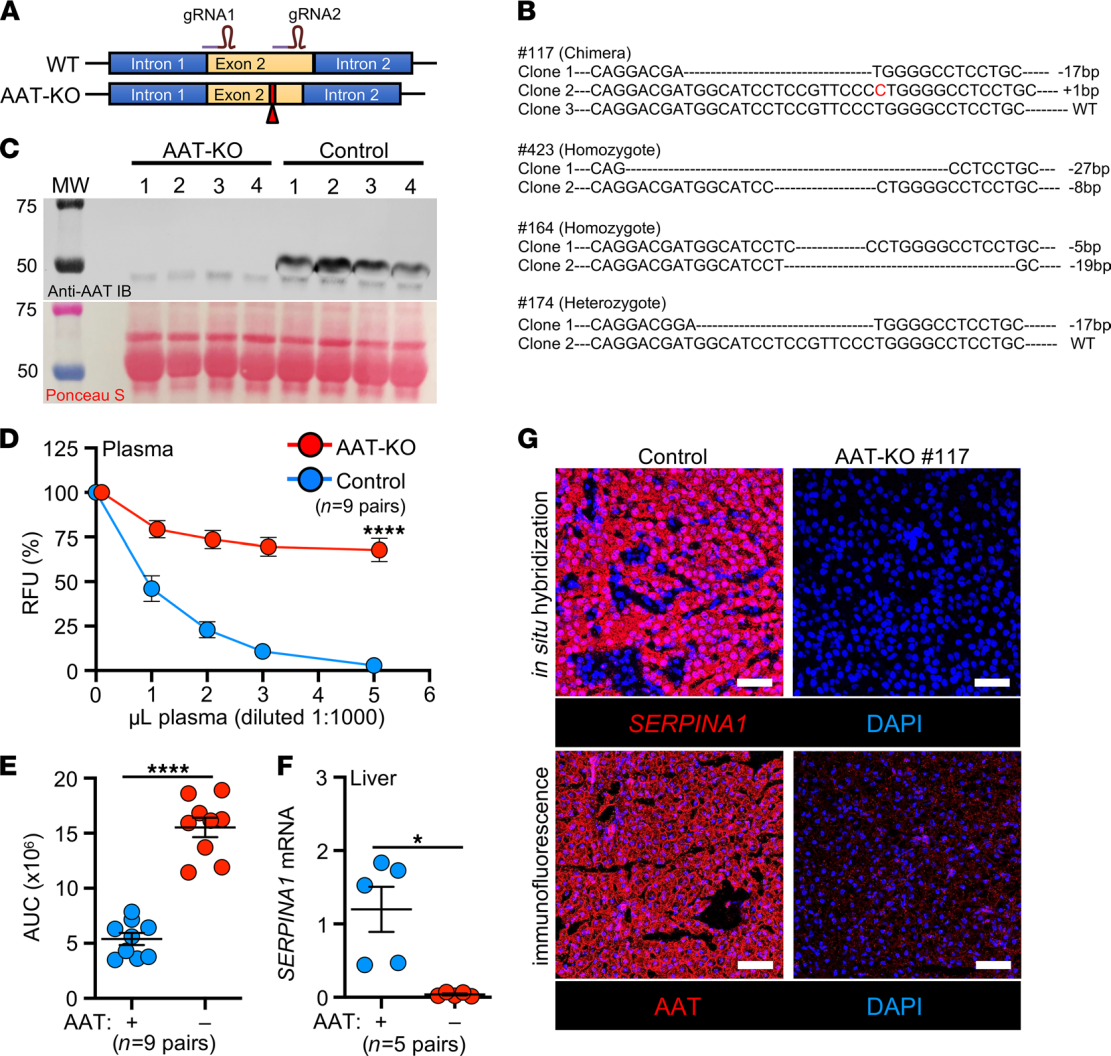
Generation and validation of an AAT-KO ferret. (**A**) Schematic for Cas9-mediated disruption of the *SERPINA1* locus in ferret zygotes. (**B**) Sequences of founders screened for expression, with the insertion and deletion polymorphisms they harbored depicted. (**C**) Western blot of plasma AAT protein in 4 AAT-KO ferrets and matched WT controls. Ponceau S–stained loading control is shown below. All draws were obtained at approximately 2 months old. (**D**) Inhibitory capacity of NE in increasing volumes of plasma from AAT-KO and control ferrets (*n* = 9 pairs; *P* value by mixed effects model, *P* = 1.6 × 10^–5^). (**E**) Quantification of NE inhibitory capacity based on AUC for each genotype in **D** (*n* = 9 pairs; *P* value by Student’s *t* test, *P* = 2.9 × 10^–7^). (**F**) Quantification of *SERPINA1* mRNA expression in liver, normalized to GAPDH (*n* = 5 pairs; *P* value by Student’s *t* test, *P* < 0.05). (**G**) Localization of *SERPINA1* transcripts and AAT protein in liver sections from a representative matched pair. Nuclei are counterstained with DAPI (blue). Scale bars: 50 μm. All graphs show mean ± SEM; some error bars are hidden by symbols. *P < 0.05; ****P < 0.0001.

We used CRISPR/Cas9 to introduce frameshift mutations in exon 2 of the ferret *SERPINA1* gene (Figure 1A, GenBank, NW_004569188). Founder animals were identified by sequencing (Figure 1B and Supplemental Table 1; supplemental material available online with this article; https://doi.org/10.1172/jci.insight.143004DS1) and were crossed to generate AAT-KO ferrets (Supplemental Table 2). Assessment of AAT protein in the plasma confirmed its absence in AAT-KO ferrets (Figure 1C). Because other proteins can act as antiproteases (9, 28), we measured the capacity of AAT-KO and control plasma to inhibit activated NE. This demonstrated a 75% reduction in antiprotease activity (Figure 1, D and E). AAT protein and mRNA transcripts were also absent in the livers of AAT-KO ferrets (Figure 1, F and G). Thus, CRISPR/Cas9-mediated targeting of *SERPINA1* produced ferrets that lacked AAT expression and reduced NE inhibition.

### Absence of AAT leads to increased lung compliance.

**Figure 2:**
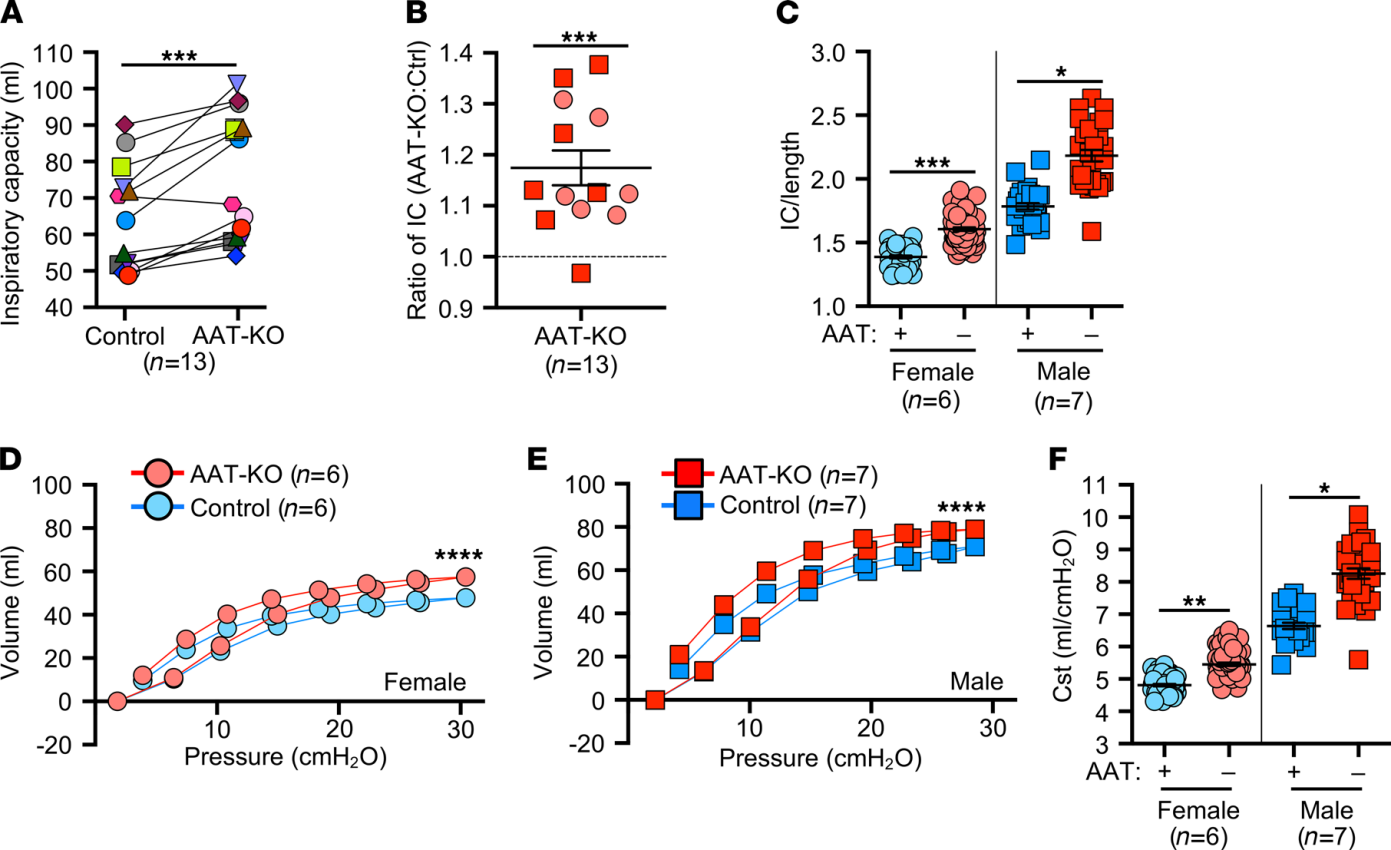
Absence of AAT leads to increased pulmonary compliance. (**A**) Inspiratory capacity (IC, mL) at 30cmH_2_O in matched pairs of AAT-KO and control ferrets in which multiple measurements were obtained during the study. Each animal is shown as 1 unique symbol to compare the average for each (*n* = 13 pairs; *P* value by mixed effects model, *P* = 0.0004). (**B**) Ratio of IC for AAT-KO to control. Each data point is the average for a given animal over its paired control (*n* = 13 animal pairs; log-transformed ratios are compared to unity by mixed effects model, *P* = 0.0001). (**C**) IC/length compared by sex and genotype; each data point is 1 measurement (*n* = 29–64 experiments in 13 paired animals; *P* value by mixed effects model, *P* = 2.158 × 10^–4^ for females and *P* = 0.021 for males). (**D** and **E**) Pressure-volume loops (PV-loops) for female (**D**) and male (**E**) ferrets of the indicated genotypes at the last experiment (*n* = 6–7 per genotype; *P* value by quadratic regression, *P* < 0.0001 for each sex). (**F**) Quasistatic compliance (Cst, mL/cmH_2_O) shown for all experiments (*n* = 28–64 experiments in 13 paired animals; *P* value by mixed effects model, *P* = 0.0027 for females and *P* = 0.032 for males). In **B**–**F**, blue indicates control ferrets and red AAT-KO. In **B**–**F**, squares indicate male ferrets and circles female. All graphs show mean ± SEM; some error bars are hidden by symbols. Data are compared as indicated within the description for each graph. *P < 0.05, **P < 0.01, ***P < 0.001, ****P < 0.0001.

We hypothesized that a lack of circulating AAT would result in spontaneous pulmonary emphysema in the absence of direct environmental insults (e.g., cigarette smoke). This concept has been difficult to study in humans (29), despite the clear association that usage of smoke tobacco products enhances the severity of lung disease in AATD patients (5, 30). To test our hypothesis, we reared a cohort of AAT-KO ferrets and age-, sex-, and size-matched controls (Supplemental Figure 1 and Supplemental Tables 2 and 3) in identical housing and assessed pulmonary mechanics over 3 years (Figure 2). The AAT-KO ferrets exhibited a larger IC than matched controls (Figure 2A and Supplemental Figure 2A). When all time points were averaged for each AAT-KO ferret and indexed to the average for the matched control, the IC was found to be approximately 116% greater (Figure 2B). As lung volumes are proportional to body height, we divided the IC by body length (IC/body length, IC/Ln), to control for large sexual dimorphism in ferret body size (Figure 2C). When sex was controlled for as an independent variable influencing lung volumes (31), the lack of AAT was found to correlate with a larger IC/Ln in both females and males. Moreover, there was a progressive increase in IC/Ln as animals aged that was accentuated in the AAT-KO ferrets (Supplemental Figure 2, B and C).

Respiratory system resistance (Rrs) was lower in female AAT-KO ferrets than controls but did not differ between genotypes in males (Supplemental Figure 2D). As expected, dynamic compliance (Crs) was higher in both male and female AAT-KO ferrets than in matched controls (Supplemental Figure 2E), whereas the opposite was true for elastance (Ers) (Supplemental Figure 2F). PV-loops at the last experimental time point (Figure 2, D and E) were shifted upward in both female (Figure 2D) and male (Figure 2E) AAT-KO ferrets. This increase in Cst shows that loss of AAT resulted in greater lung compliance (Figure 2F).

### AAT-KO ferret lungs develop fixed airflow limitations.

While emphysema is caused by an increase in pulmonary compliance, in humans the condition is diagnosed by the presence of a fixed airflow limitation (32, 33). This is measured by quantifying the ratio of the volume of air exhaled in 1 second (forced expiratory volume, FEV) to the total volume of air exhaled (forced vital capacity, FVC). In COPD the FEV_1_:FVC ratio is <0.7 (33, 34). We designed a device that performs forced expiratory maneuvers in ferrets (Supplemental Figure 3A). Whereas FEV_1_ is used in humans to identify airflow limitations, the equivalent time point (FEV_X_) was not known in ferrets. To identify FEV_X_, we first determined the FEV_X_:FVC at which control ferrets (*n* = 5) remained >0.7. Our data suggest that FEV_0.4_ in ferrets may be analogous to human FEV_1_ (Supplemental Figure 3, B and C). Notably, this is close to what is used in human infants (FEV_0.5_) (35, 36).

**Figure 3:**
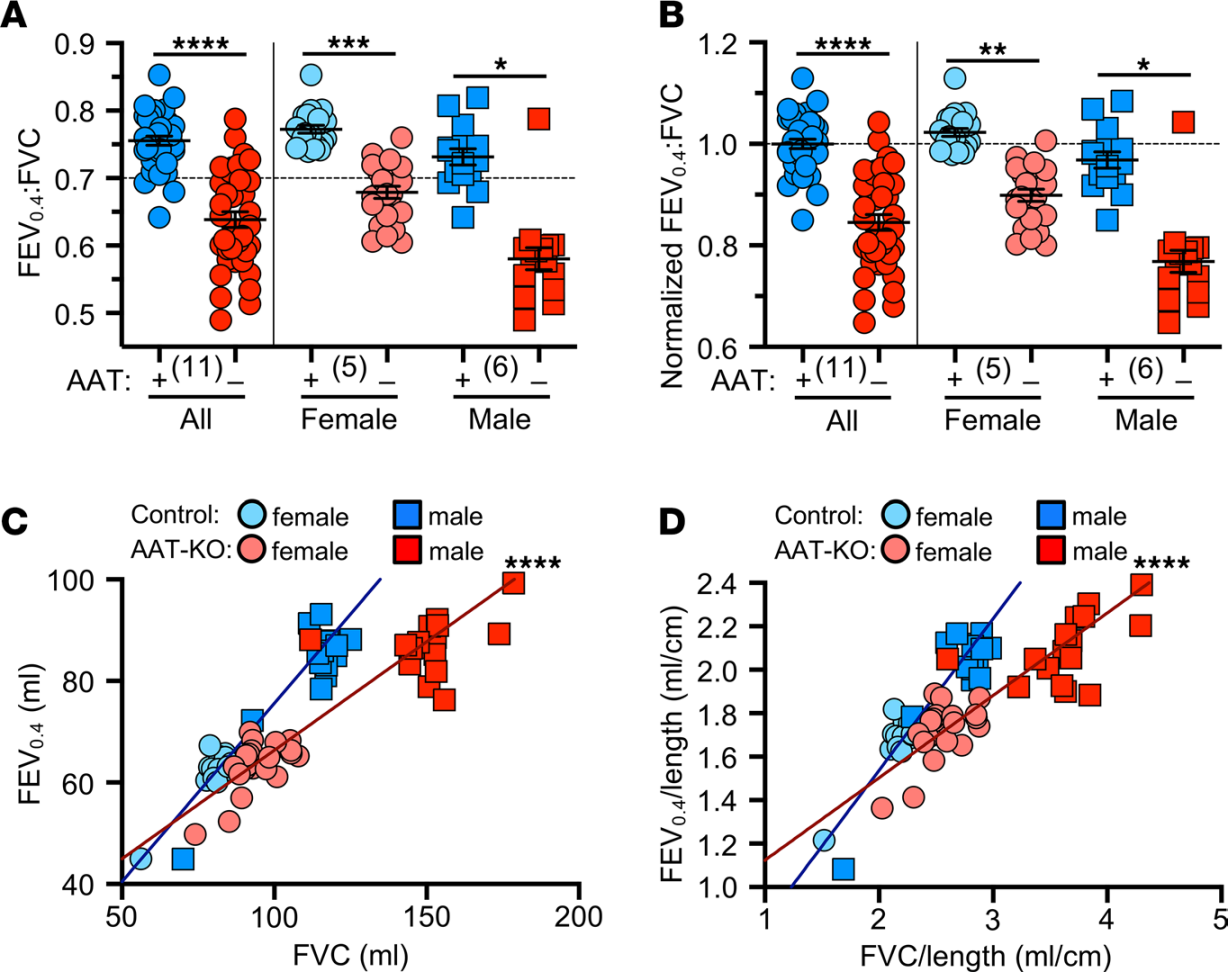
Absence of AAT is sufficient to cause airflow limitation and obstruction. (**A**) Ratio of FEV_0.4_ to FVC for AAT-KO and control ferrets, broken down by sex to the right of the vertical line (*n* = 35–38 experiments in 11 paired animals; *P* value by mixed effects model, *P* = 0.000026 for both sexes together, *P* = 0.00029 for females, and *P* = 0.048 for males). (**B**) Normalized FEV_0.4_:FVC ratio where controls are set to 1, broken down by sex to the right of the vertical line (*n* = 35–38 experiments in 11 paired animals; *P* value by mixed effects model, *P* = 0.000031 for both sexes together, *P* = 0.0032 for females, and *P* = 0.048 for males). (**C** and **D**) FEV_0.4_ plotted against FVC for each animal as (**C**) raw volume and (**D**) normalized by body length (*n* = 35–38 experiments in 11 paired animals; *P* value by mixed effects model fit to each genotype to compare slopes; *P* = 9.362 × 10^–6^ for **C** and *P* = 4.621 × 10^–6^ for **D**). (**A** and **B**) Mean ± SEM with the biological *n* in parentheses; the same data are presented in **C** and **D**. *P < 0.05; **P < 0.01; ***P < 0.001; ****P < 0.0001.

We then measured the FEV_0.4_:FVC ratio of AAT-KO and control ferrets (*n* = 11 pairs). As predicted, airflow obstruction was present in most AAT-KO ferrets, as indicated by an FEV_0.4_:FVC ratio < 0.7 (Figure 3A). To determine whether this was progressive, we analyzed the FEV_0.4_:FVC in ferrets over time. This did not reveal a decline, although this could be due to the limited sample size (Supplemental Figure 4). Given that FEV_0.4_ did not result in a FEV_0.4_:FVC ratio of >0.7 for all control males, it is possible that due to sexual dimorphism the ratio should be adjusted based on ferret size. To control for size, we normalized the FEV_0.4_:FVC ratio to the average for all control ferrets and found that the results were similar (Figure 3B). The FEV_0.4_ was compared to the FVC for each ferret experiment so that differences in lung volumes were controlled for directly. This relationship was perturbed in the AAT-KO ferrets, because they exhaled less volume for a given FVC (Figure 3C), and the same was true when the FEV_0.4_ and FVC were each normalized to Ln (Figure 3D).

### Airflow obstruction in AAT-KO ferrets is driven by emphysema.

**Figure 4:**
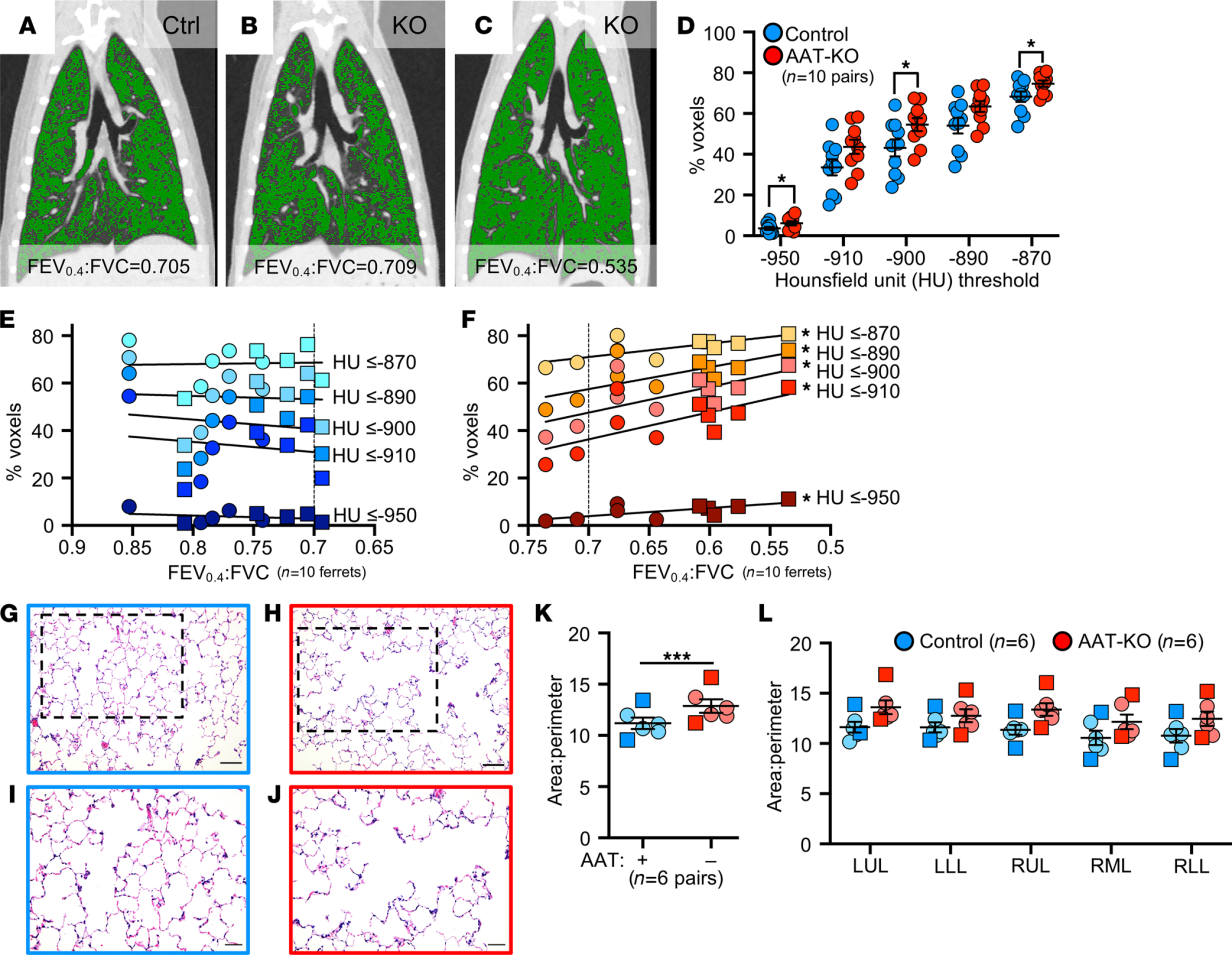
Emphysema drives airflow obstruction in AAT-KO ferrets. (**A**–**C**) Representative coronal CT reconstructions from control (**A**) and AAT-KO (**B** and **C**) ferrets with voxels at or below Hounsfield units (HU) of –900 pseudocolored in green. FEV_0.4_:FVC is indicated below each panel. (**D**) QCT analysis of AAT-KO and control ferrets at total lung capacity (TLC, airway pressure = 25cmH_2_O) reveals increased percentage of lower density voxels at various standard HU thresholds (*n* = 10 matched ferret pairs; *P* value by multivariate linear regression for all measures together, *P* = 0.025. Also shown are Student’s *t* tests for each measurement where *P < 0.05, specifically: –950, –900, and –870 HU. (**E** and **F**) Percentage of voxels at or below each indicated HU threshold plotted against the FEV_0.4_:FVC ratio for each control (**E**) and AAT-KO (**F**) ferret. (*n* = 10 matched ferret pairs; *P* value by linear regression model, *P* < 0.05 for all AAT-KO.) (**G**–**J**) Representative sections of lung from control (**G** and **I**) and AAT-KO (**H** and **J**) ferrets stained with H&E. Boxed regions in **G** and **H** are enlarged in **I** and **J**, respectively. Scale bars in **G** and **H** represent 100 μm and in **I** and **J** represent 50 μm. (**K**) Summary of alveolar area-to-perimeter ratio in inflation-fixed lungs with all lobes averaged for each animal (*n* = 6 ferrets from each genotype; *P* value by paired Student’s *t* test, *P* < 0.001). (**L**) Data from **K** are shown for each lobe. In **E**, **F**, **K**, and **L**, squares = males and circles = females. Graphs in **D**, **K**, and **L** show mean ± SEM. *P < 0.05, ***P < 0.001. LUL, left upper lobe; LLL, left lower lobe; RUL, right upper lobe; RML, right middle lobe; RLL, right lower lobe.

To confirm anatomical lesions consistent with emphysema, we used in vivo quantitative computed tomography (QCT) imaging and analyzed airspace morphometry at the time of sacrifice (Figure 4 and Supplemental Tables 4 and 5). To this end, QCT imaging was performed during an inspiratory hold (Figure 4, A–C; TLC, 25cmH_2_O) and processed using customized QCT software to calculate the percentage of low-attenuation voxels, indicating emphysematous lung, at or below prespecified HU thresholds identified in participants with COPD (37–40). The proportion of low-density voxels was greater in AAT-KO ferrets than controls at TLC (Figure 4D). To map airflow limitation to emphysema on inspiratory CT scans, the percentage of voxels at or below each of the HU thresholds was plotted against the ferret’s FEV_0.4_:FVC ratio (Figure 4, E and F, and Supplemental Figure 5). In AAT-KO ferrets, a greater degree of airflow obstruction (reduced FEV_0.4_:FVC ratio) was correlated with a greater proportion of low-attenuation voxels. This relationship is quantified by the slope of the best fit line, and we observed a decreasing slope in the AAT-KO ferrets as the threshold was lowered from –910 to –870 HU (Figure 4F), which is what would be predicted if emphysematous lung were driving the airflow obstruction, as including less dense regions reduces the correlation (slope). To corroborate these findings with histopathology, we examined lung sections from AAT-KO and control ferrets fixed at 25cmH_2_O (Figure 4, G–L). Comparison of the surface area to perimeter (SA/P) ratio as a measure of enlargement determined that airspaces were 115% greater in AAT-KO ferrets than matched controls (Figure 4K). Notably, this is similar to the IC ratio for AAT-KO to controls (116%; Figure 2B) and to the difference between FEV_0.4_:FVC ratios when normalized (0.84; Figure 3B). Collectively, these data verify that AAT deficiency in the ferret results in pulmonary emphysema.

### BAL proteome of AAT-KO ferrets suggests enhanced inflammation.

**Figure 5:**
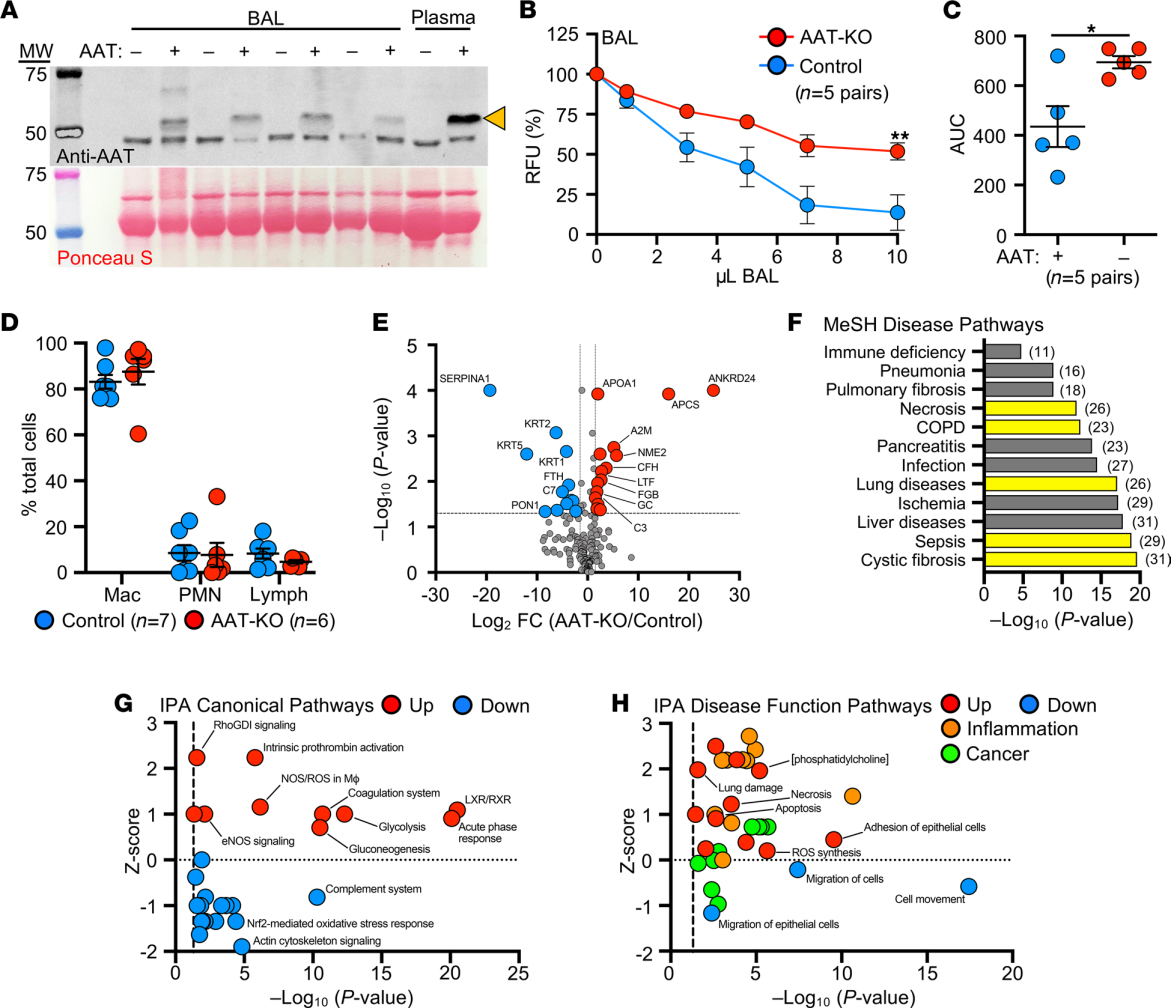
BAL from AAT-KO ferrets has reduced NE inhibitory capacity, and secreted proteome suggests enhanced inflammation and lung damage pathways. (**A**) Western blot of BAL from AAT-KO (dash) and matched control ferrets (plus); arrowhead indicates AAT band in plasma as positive and negative controls. Ponceau-stained blot as loading control. (**B**) NE inhibitory capacity of increasing volumes of BAL from AAT-KO and matched control ferrets (*n* = 5 pairs; *P* value by mixed effects model, *P* = 0.005). (**C**) Quantification of NE inhibitory capacity by calculating the AUC in **B** (*n* = 5 pairs; *P* value by Student’s *t* test, *P* = 0.0297). (**D**) Differential of cell types in BAL from control and AAT-KO ferrets (*n* = 6–7 ferrets; *P* value by Student’s *t* test, *P* > 0.3 for each cell type). Mac, macrophages; PMN, polymorphonuclear leukocytes. (**E**) Volcano plot of more than 200 BAL proteins from 7 age-matched AAT-KO and control ferrets (red circles, upregulated; blue circles, downregulated; gray circles, indeterminate or not statistically significant; *n* = 7 matched pairs; *P* value by Scaffold software *t* test). (**F**) List of selected significant disease pathways discovered using MeSH analysis of the BAL proteome. Yellow bars denote disease-associated hits that are relevant to lung diseases, and numbers in parentheses indicate number of proteins found in the pathway. (**G** and **H**) IPA performed on proteomics data where (**G**) canonical and (**H**) disease function pathways are plotted against *z* score and –log_10_(*P* value). In both panels, red indicates upregulated and blue downregulated pathways by *z* score; in **H** inflammation-related pathways are in orange and cancer-related pathways are in green. Graphs in **B**–**D** show mean ± SEM. *P < 0.05, **P < 0.01. FC, fold change.

Given that lung disease was progressive in our AAT-KO ferret cohort (Supplemental Figure 2C), we hypothesized that ongoing inflammation plays a prominent role in pathogenesis. We tested this by collecting BAL from AAT-KO and controls of similar ages (~400 days old; Supplemental Table 4) and verified that AAT was absent and that this led to reduced capacity to inhibit NE (Figure 5, A–C). Examination of the cell types retrieved in the lavage did not suggest a neutrophilic or lymphocytic predominance in the AAT-KO ferrets (Figure 5D). Reasoning that unopposed protease activity would alter the airway proteome, we performed quantitative liquid chromatography–tandem mass spectrometry on BAL as an alternative measure of airway inflammation (Figure 5E and Supplemental Table 6) (24, 25, 28). The secretory proteome of AAT-KO ferrets was characterized by increased APOA1, APCS, A2M, ENO1, and fibrinogen (FGB and FGG), as well as decreased TF and VIM, a profile that is concordant with proteomic investigations comparing patients with COPD and healthy humans (41–43). Although reduced complement levels in COPD BAL have been described in other studies, namely C1q, C2, C3, C4b, and fibronectin (41), we did not find a consistent reduction in our experiments. This may have been limited by statistical power. A notable finding within our BAL proteomics was that paraoxonase-1 (PON1) was 300-fold lower in AAT-KO ferret BAL than in controls (Figure 5E). This protein has been observed to be reduced in an ovalbumin-induced model of asthma where overexpression was associated with improved airway inflammatory state and limited remodeling (44). Moreover, expression of PON1 is protective against pseudomonal infection (45), which suggests that downregulation of PON1 in the BAL of AAT-KO ferrets may be of interest in future studies of airway infection.

Pathway analysis of proteomics data based on Medical Subject Headings (MeSH) identified features of lung diseases (Figure 5F and Supplemental Table 7), including cystic fibrosis, COPD, and pneumonia. Furthermore, Ingenuity Pathway Analysis (IPA) provided evidence of liver X receptor and retinoid X receptor activation and acute phase response upregulation (Figure 5G and Supplemental Table 8) along with activated inflammatory and lung damage pathways (Figure 5H and Supplemental Table 9). Consistent with structural lung disease being caused by impaired remodeling, this pathway analysis indicates the presence of inflammatory lung disease with muted cellular migration in the context of apoptosis, damage, and necrosis. The activation and repression of these pathways suggest that inflammatory stimuli can accelerate the development of lung disease in AAT-KO ferrets. This has been suggested in cohort studies of patients with AATD, who were more likely to have respiratory symptoms if they were exposed to tobacco smoke or had a respiratory infection during childhood (46).

Given these findings, we conducted an abbreviated inflammatory challenge in a cohort of young AAT-KO ferrets (*n* = 4 pairs/group) using intratracheally administered LPS in sequential doses (2 mg/kg in 2 mL saline at 60 days old and half-dose 14 days later, Supplemental Figure 6A) to show that this could be used to worsen disease severity in the AAT-KO model. Indeed, LPS challenge to AAT-KO ferrets demonstrated a significantly decreased FEV_0.4_:FVC, increased pulmonary compliance, and enlarged airspaces at the time of sacrifice (Supplemental Figure 6, B–F), as compared with vehicle-challenged AAT-KO ferrets. By contrast, there was no difference in these parameters between control LPS and vehicle-challenged ferrets.

### Insertion of the Z-allele into the ferret SERPINA1 locus leads to both liver and lung disease.

**Figure 6:**
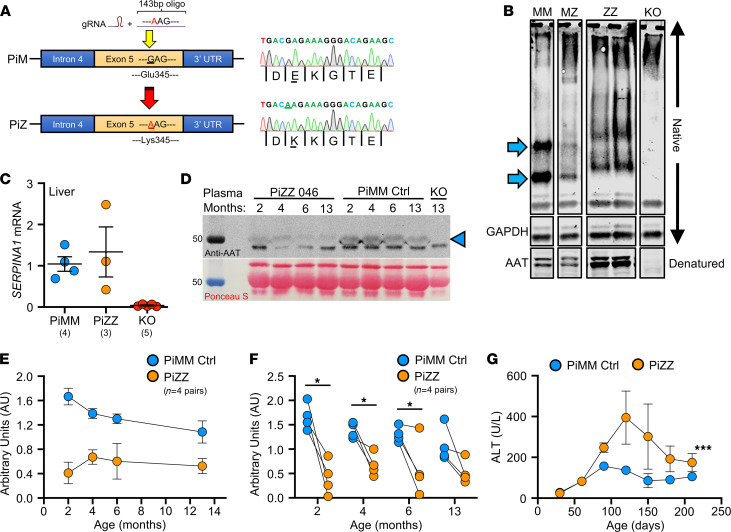
Generation of a PiZZ ferret model of AATD and characterization of hepatic disease. (**A**) Schematic depiction of CRISPR/Cas9-mediated Z-allele knockin (with donor oligo) in exon 5 of the *SERPINA1* locus. Founders were confirmed by Sanger sequencing (at right). (**B**) Representative lanes from native PAGE gel performed on liver tissue lysates showing change in band migration of the PiZ protein. Arrows mark the PiMM (MM) control bands that are shifted in PiZZ (ZZ) samples and absent in the AAT-KO sample. PiMZ (MZ) shows a combined pattern. GAPDH is shown as loading control for the native PAGE. Denatured samples were separated by SDS-PAGE and probed for AAT at the bottom of the panel. (**C**) mRNA expression of *SERPINA1* in PiZZ ferret liver tissue compared with PiMM controls and AAT-KO nulls (*n* = 3–5 ferrets/group as indicated). (**D**) Western blot of plasma from a representative PiZZ ferret and age-matched PiMM control showing change in circulating AAT over time (AAT band marked by arrowhead). Timed blood draws in PiZZ and PiMM ferrets at 2, 4, 6, and 13 months old; AAT-KO ferret drawn at 13 months old as negative control. Below is the Ponceau stain used for loading control. (**E**) Densitometric quantification of plasma AAT (normalized to Ponceau band) over time in 4 pairs of PiZZ and PiMM control ferrets. (**F**) Comparison of plasma AAT at each blood draw (*n* = 4 pairs at each time point; *P* value by paired Student’s *t* test, *P < 0.05). (**G**) Levels of plasma ALT over time in PiZZ and PiMM control ferrets (*n* = 2–5 ferrets at each time point; *P* value by mixed effects model, ***P = 0.00014). In **C** and **E**–**G**, blue = PiMM controls, yellow = PiZZ, and in **C**, red = AAT-KO. All graphs show mean ± SEM; some error bars are hidden by symbols. *P < 0.05; ***P < 0.001.

In humans, the most frequent Z-allele mutation is an E342K substitution in exon 5 of *SERPINA1*; in ferrets, the analogous variant is E345K. The amino acids surrounding E345 (VLTIDEKGTEA) are 100% conserved between human and ferret, suggesting similar folding disturbances may occur between the protein orthologs with an E345K mutation. Thus, we targeted exon 5 using Cas9/guide RNA (gRNA) complex and supplied a donor oligo encoding Glu345Lys/E345K (Figure 6A). Sequencing identified the resultant founder as homozygous E345K (PiZZ), and it was bred to expand the line (Supplemental Tables 10 and 11).

As the Z-allele is characterized by accumulated protein within hepatocytes (16, 47), we harvested liver lysates from 4- to 7-month-old PiZZ ferrets and compared them with PiMM (WT controls), PiMZ (heterozygous), and AAT-KO using native PAGE to assess for the presence of polymerized protein (Figure 6B and Supplemental Figure 7). Whereas PiMM controls had 2 distinct bands (blue arrows), in PiZZ tissue these bands were shifted upward and less distinct, suggesting a greater variety of sizes within the sample consistent with polymerization. The PiMZ ferret tissue exhibited a composite appearance of both PiM and PiZ, and the AAT-KO ferret samples lacked any band patterns. When these samples were denatured and separated by SDS-PAGE, there appeared to be a greater amount of AAT in the PiZZ tissues, which would be expected due to retention within hepatocytes, although enhanced *SERPINA1* expression could also explain this finding (15). To assess this possibility, we measured mRNA in PiZZ and PiMM control tissue and found that it was similar (Figure 6C). As AAT-PiZ is expressed at similar levels but aggregates within hepatic tissues, we expected that there would be less AAT in circulating plasma. To evaluate the reduction in protein due to the AAT-PiZ mutation, we compared plasma AAT levels over time in a cohort of PiZZ ferrets and compared this with matched PiMM controls (Figure 6, D–F). These PiZZ ferrets had on average approximately 40% less AAT in the plasma as compared with controls. Given that PiZ-AAT has folding defects that alter migration through the native PAGE separation, we suspected that intracellular accumulation would result in hepatocellular injury. To test this, we measured alanine transferase (ALT), an enzyme released from hepatocytes that indicates hepatocellular injury, over time in PiZZ and PiMM control ferrets (Figure 6G). Whereas at 60 days, PiZZ and PiMM control ferrets had similar ALT levels, by 120 days (4 months old), we observed that PiZZ levels peaked 4-fold higher than controls. This ALT elevation subsequently stabilized at about twice that of PiMM controls. Collectively, these data suggest that hepatocytes in the PiZZ ferrets express similar levels of hepatic *SERPINA1* mRNA and that the mutant Z protein appears to form intrahepatic polymers that lead to hepatocellular injury and reduced circulating AAT. Evaluation of diastase-resistant periodic acid–Schiff^+^ aggregates in hepatic sections of these 4- to 7-month-old animals demonstrated no differences between genotypes (data not shown), suggesting liver disease was at a relatively early stage. Whether this hepatocellular injury leads to chronic liver disease remains to be determined, as animals in the study were still relatively young.

**Figure 7:**
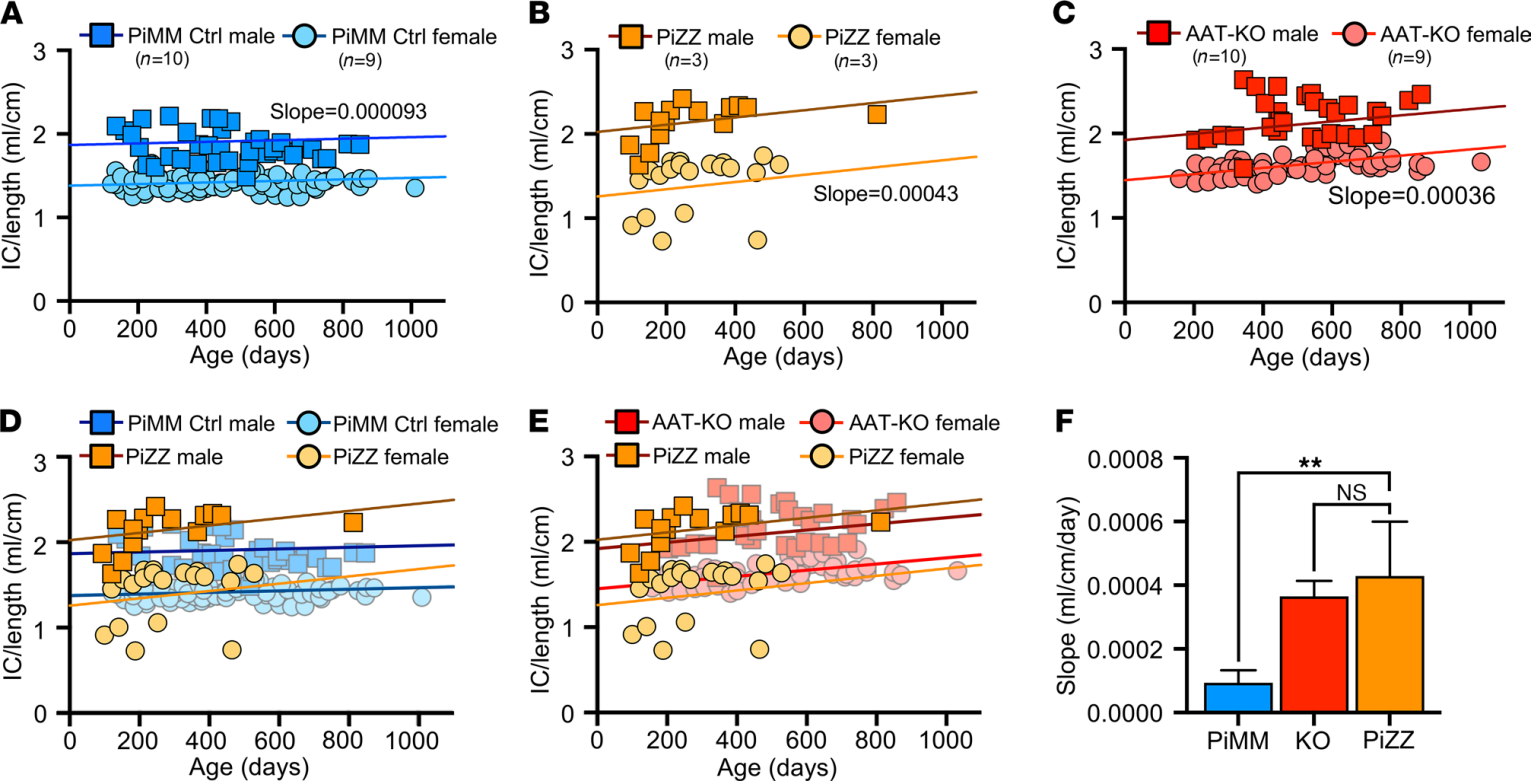
PiZZ ferrets develop age-dependent increase in pulmonary compliance. (**A**–**C**) IC/Ln shown over time for PiMM control ferrets (**A**), PiZZ ferrets (**B**), and AAT-KO ferrets (**C**). A mixed effects model was fit to each genotype, with a separate intercept for the sex. The slope of PiMM controls (**A**) is 0.000093 mL/cm per day, for PiZZ ferrets (**B**) it is 0.00043 mL/cm per day, and in AAT-KO ferrets (**C**) it is 0.00036 mL/cm per day (*n* = 37 experiments in 6 PiZZ animals vs. *n* = 112 in 19 PiMM control animals vs. *n* = 95 experiments in 13 AAT-KO animals). (**D** and **E**) IC/Ln for PiZZ ferrets is compared with data sets for PiMM controls (**D**) and AAT-KO ferrets (**E**). (**F**) Slope of the mixed effects model fit for each genotype is compared (*P* value by mixed effects model interaction, *P* = 0.0078 for PiZZ vs. PiMM controls and *P* = 0.67 for PiZZ vs. AAT-KO). In all panels, blue = PiMM controls, orange = PiZZ, and red = AAT-KO. Squares are males and circles female. In **F**, the bar represents the mean ± SEM for the slope as calculated by a mixed effects model. **P < 0.01.

**Figure 8:**
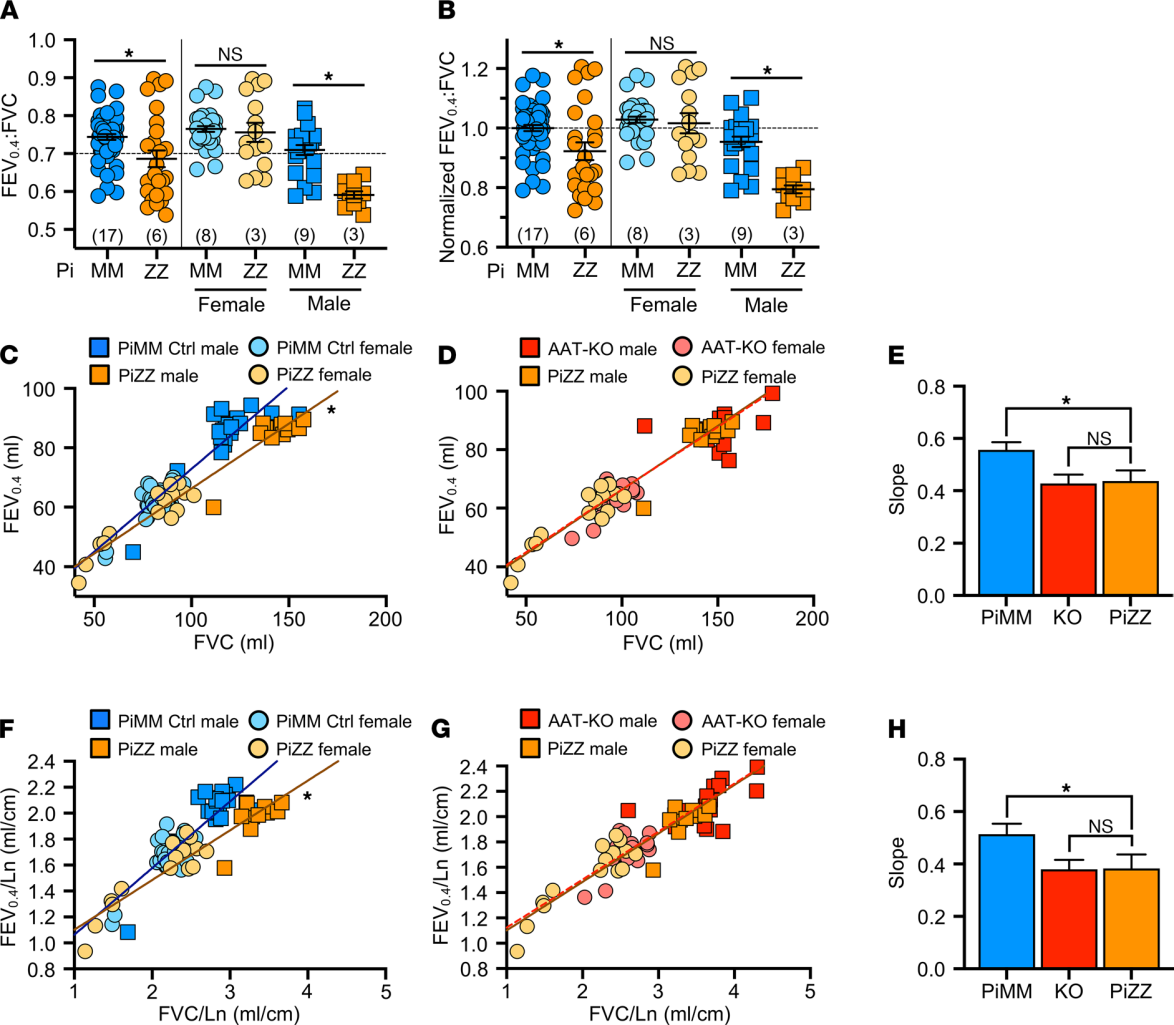
PiZZ ferrets develop airflow obstruction. (**A**) FEV_0.4_:FVC ratio for PiZZ and PiMM controls, separated by sex to the right of the vertical line (*n* = 26–58 experiments in 6 PiZZ and 17 PiMM controls; *P* value by mixed effects model, *P* = 0.026 for both sexes, *P* = 0.54 for females, and *P* = 0.016 for males). (**B**) Normalized FEV_0.4_:FVC ratio with controls set to 1, separated by sex to the right of the vertical line (*n* = 26–58 experiments in 6 PiZZ and 17 PiMM controls; *P* value by mixed effects model, *P* = 0.016 for both sexes, *P* = 0.45 for females, and *P* = 0.013 for males). (**C** and **D**) FEV_0.4_ plotted against FVC for each animal where (**C**) compares PiZZ and PiMM controls and (**D**) compares PiZZ and AAT-KO ferrets (*n* = 26–58 experiments in 6 PiZZ and 17 PiMM controls; *P* value by mixed effects model fit to each genotype, *P* = 0.014 for **C** and *P* = 0.25 for **D**). (**E**) Slope of the mixed effects model fit for each genotype in **C** and **D** are compared (*P* value by mixed effects model interaction, *P* = 0.014 for PiZZ vs. PiMM controls and *P* = 0.25 for PiZZ vs. AAT-KO). (**F** and **G**) FEV_0.4_ plotted against FVC, normalized by body length (FEV_0.4_/Ln vs. FVC/Ln), for each animal, where (**F**) compares PiZZ and PiMM controls and (**G**) compares PiZZ and AAT-KO ferrets (*n* = 26–58 experiments in 6 PiZZ and 17 PiMM controls; *P* value by mixed effects model fit to each genotype, *P* = 0.026 for **F** and *P* = 0.25 for **G**). (**H**) Slope of the mixed effects models fit to genotype in **F** and **G** are compared (*P* value by mixed effects model interaction, *P* = 0.026 for PiZZ vs. PiMM controls and *P* = 0.25 for PiZZ vs. AAT-KO). In all panels, blue = PiMM controls, orange = PiZZ, and red = AAT-KO. Squares are males and circles female. In **E** and **H**, the bar represents the mean ± SEM for the slope calculated by mixed effects model. *P < 0.05.

Having shown that PiZZ ferrets are AAT deficient, we asked if they would develop obstructive lung disease similar to the AAT-KO model. To determine this, we followed the lung function of 6 PiZZ ferrets (3 male, 3 female) and compared them with all PiMM controls in the study (Figures 7 and 8 and Supplemental Figure 8, A–D). As inspiratory capacity normalized by body length (IC/Ln) provides information about lung compliance and our custom maneuver can accommodate larger animal sizes than the PV-loops, we determined the change in this measure over time in PiMM control ferrets (slope = 0.000093, Figure 7A), PiZZ ferrets (slope = 0.00043, Figure 7B), and AAT-KO ferrets (slope = 0.00036, Figure 7C). When we compared PiZZ with PiMM control (Figure 7D) and AAT-KO (Figure 7E) ferrets, we observed that PiZZ developed increased pulmonary compliance (i.e., IC/Ln) as they aged that is similar to that of AAT-KO ferrets (Figure 7F). Reasoning that this increased compliance would lead to airflow obstruction in the PiZZ ferrets as in the AAT-KO line, we determined the FEV_0.4_:FVC ratio in the PiZZ cohort and compared this with the FEV_0.4_:FVC ratio of our PiMM ferrets (Figure 8, A and B). In the PiZZ cohort we observed that males developed airflow limitation earlier in life than female PiZZ ferrets (Supplemental Figure 8E); while this differed from the AAT-KO ferret model (Supplemental Figure 4), it may be due to the younger age of the PiZZ animals in our study. When the FEV_0.4_ was plotted against FVC for each experiment, PiZZ ferrets had a lower FEV_0.4_ than PiMM controls (Figure 8C), and this matched the FEV_0.4_ of the AAT-KO ferrets (Figure 8D). The slope of this line was identical for PiZZ and AAT-KO ferrets and was significantly lower than PiMM controls (Figure 8E). This finding was unchanged when lung volumes or capacities were adjusted for body length (Figure 8, F–H). These data indicate that the PiZZ ferret develops increased pulmonary compliance and airflow limitation, which is more severe in male ferrets than female and occurs in the absence of any directed insult or challenge. A similar finding regarding the increased risk for lung function deterioration in males over females has been observed in cohorts of humans with AATD who are lifelong nonsmokers (48).

## Discussion

Our longitudinal experiments using the AAT-KO ferret demonstrate that: (a) the loss of AAT results in emphysema as defined by increased lung compliance (Figure 2), expiratory flow limitation (Figure 3), and enlarged alveolar spaces (Figure 4); and (b) this occurs in the absence of direct insult to the lungs and under controlled conditions. These findings confirm that AAT is required to prevent spontaneous emphysema in the ferret, which mirror and expand upon the results reported in the SerpinA1a-e–KO mouse (20).

Our studies in the AAT-KO ferret indicate that lung compliance is elevated at the youngest age at which we measured pulmonary mechanics (Supplemental Figure 2, B and C). This suggests that the loss of AAT may affect humans at an early age. More sensitive measures of peripheral airway disease, specifically using multiple breath nitrogen washout techniques, have found subtle ventilatory inhomogeneity in PiZZ adolescents (49), supporting the notion that loss of AAT function results in ongoing subclinical proteolytic lung damage despite normal spirometry. The finding that male AAT-KO and PiZZ ferrets appeared to have a greater increase in both pulmonary compliance and airflow obstruction than females is in keeping with reports that AATD males may be more susceptible to declining pulmonary function (48, 50). This finding in the AATD ferret is limited by numbers, but the model provides a means to investigate this finding in greater detail. We found that AATD ferrets had features of emphysema at adolescence, which is similar to the duration of cigarette smoke exposure necessary to develop chronic bronchitis (23). We also found that an inflammatory insult with LPS led to increased airspace size and enhanced airflow obstruction in AAT-KO ferrets (Supplemental Figure 6). These outcomes align with studies of patients with AATD suggesting that childhood exposure to smoke, and perhaps airway infections, predispose to later respiratory symptoms (46).

Our proteomics analysis of BAL is also consistent with previous reports that AAT has immunomodulatory roles (51–54), given the enhanced inflammatory pathways in the lung (Figure 5) and evidence of extrapulmonary involvement (Supplemental Figure 9). The AAT-KO ferret model provides an opportunity to test these hypotheses prior to the onset of lung disease. Moreover, our BAL proteomics analysis showed that the quantity of alpha-2 macroglobulin, a recognized protease inhibitor (55, 56), increased in the AAT-KO animals as has been observed in serum from young individuals with AATD (57). Alpha-2 macroglobulin may have counteracted the loss of AAT to some degree, as we did find this protein within our BAL samples, which also suggests ongoing inflammation. Finally, our experiments demonstrate that the PiZZ knockin exhibits altered folding of the AAT protein that accumulates in liver tissues (Figure 7) and that this accumulated protein leads to hepatocellular injury. Reduced circulating AAT in the PiZZ ferret also resulted in altered lung function. The PiZZ ferret may be useful in studying how liver disease interacts with lung health (58).

### Study limitations.

This study has several limitations. First, although AAT-KO, PiZZ, and control ferrets were kept in the same facilities, they could not always be cohoused, and this could lead to differential exposure to environmental stimuli. However, this exposure was likely more controlled than that experienced by a human with AATD. That most of our ferrets were studied over more than a calendar year provides similar exposure to respiratory viruses as well, which are well known to trigger episodes of lung disease exacerbation and to which the ferret is highly susceptible (24, 59). Second, our pulmonary function test system for ferrets is still in the early stages of use, and the FEV_X_ time point for therapeutic testing may need more refinement. This is particularly important for studies of the domestic ferret, which has such dramatic sexual dimorphism. We attempted to mitigate this issue by matching control to AAT-KO ferrets by sex, age, and body size. In addition, for our analyses, we indexed measurements of lung volume and capacity measures to the body length of each ferret (analogous to height in humans). Although this approach does not eliminate the need for an improved understanding of ferret pulmonary physiology in developing translational therapies, until more is known, the methods of indexing to subject size can easily be adopted by the scientific community. Third, in demonstrating airflow obstruction in the AAT-deficient ferret lung, we did not rule out the role of airway hyperreactivity, because we did not assess the response to a bronchodilator. However, airway hyperreactivity would not influence our QCT or alveolar morphometry data, and these are clearly consistent with emphysema.

### Study strengths.

The strengths of our study are as follows. First, we provide a wealth of longitudinal data on a large-animal model of genetic lung disease that shares key pathophysiology with COPD, which is the third leading cause of death in the United States. Our model complements a recent smoke exposure ferret model of chronic bronchitis (23); together these models may provide much needed insights into how AAT insufficiency caused by a single Z-allele mutation increases the risk of lung disease in users of tobacco products (60). Future experiments involving a comparison between the homozygous AAT-KO and PiZZ ferrets to heterozygous ferrets of each allele would allow us to determine if a lower level of circulating AAT or the presence of the Z-allele itself is implicated in disease pathogenesis. Second, we have developed and report a full translational platform for the study of pulmonary pathophysiology in ferrets. This includes commonly used clinical tests (e.g., pulmonary function tests, QCT, and bronchoscopy) that are expected to shed light on the efficacy of therapies over time. Third, the substantial sexual dimorphism observed in our model of lung disease provides an avenue of future study as to how this may influence and interact with disease pathogenesis and therapeutic development. Last, the PiZZ ferret model serves as a valuable addition to the AAT-KO model in that it develops hepatic injury consistent with that observed in human patients, and it allows for the study of both gene therapy and editing strategies in a relevant model of lung and liver disease. These 2 large-animal models of AATD are valuable additions to the cadre of rodent models that have been developed through genetic or environmental means (17, 18) to reduce circulating AAT and develop emphysema.

### Similarities to human disease.

Comparing our ferret models of AATD with that of human disease, we observe both similarities and differences. Reduction (PiZZ) or absence (AAT-KO) of circulating AAT in the ferret led to airflow obstruction, but only the PiZZ ferret developed hepatocellular injury, which is similar to disease observed in rare AAT-null and PiZ humans (5, 61). Lower lobar predilection of disease that is classically seen in humans with AATD was not evident in AAT-KO ferrets, which may be due to the differential effect of gravity on the horizontally oriented ferret lungs or to the archetypal basal emphysema in AATD not being as ubiquitous as previously thought (62). AAT-null humans develop clinically evident lung disease by the third decade of life (63). This age maps closely to when the AAT-KO ferret exhibits increased lung compliance (IC/Ln), at 2 years of age (approximately 25% of expected life span in the domestic ferret). In the PiZZ ferret, we determined that circulating AAT was reduced to 40% of PiMM control levels, which contrasts with the human PiZZ condition, where there is 14%–20% of the normal level (64–66). The comparatively larger liver in the ferret may explain some of this difference (4% of body weight vs. 2% in humans). We observed a transient elevation of ALT in young PiZZ ferrets similar to what is seen in about 70% of children with PiZZ (61, 67). There was no elevation in bilirubin or evidence of histological liver disease in 2-year-old PiZ//KO compound heterozygous ferrets (Supplemental Figure 7). Both the relatively small size of our cohort of PiZZ ferrets and the relatively young age of the available animals limit further comparisons between the ferret model and the human disease.

### Questions for the future.

Although the disease course of AATD in patients following diagnosis is well characterized, many questions about this condition remain: (a) When does disease begin, and how does it manifest initially? (b) When is the best time to intervene? (c) What roles beyond its antiprotease activity does AAT play? (d) What kind of genetic therapy (insertion versus editing) is best suited for this complex gain- and loss-of-function disease? and (e) Is gene therapy sufficient to rescue a decline in lung function once disease has set in? The ferret models described here and the suite of translational assays we have developed (pulmonary function tests, QCT, bronchoscopy) may provide the scientific community a means to answer these questions.

## Methods

### Animals

Domestic ferrets were obtained from Marshall Farms and housed under controlled temperature (20°C–22°C) with a 16-hour light/8-hour dark cycle and ad libitum access to water and diet. Expansion of cloned founders by breeding was conducted at Marshall Farms, while zygote editing to generate ferrets homozygous and heterozygous for either the knockout or Z-allele was performed at the University of Iowa. Controls were age-, sex-, and size-matched WT ferrets and were kept in the same facilities to control for exposures (Supplemental Figure 1 and Supplemental Tables 1–3, 10, and 11).

### gRNA design and in vitro validation

Single-guide RNAs (sgRNAs) were designed using CRISPOR (http://crispor.tefor.net) to target exon 2 (for *SERPINA1* gene disruption, AAT-KO) or exon 5 (for Z-allele single–amino acid substitution encoding the E345K mutation) of the ferret *SERPINA1* gene (GenBank, NW_004569188) and obtained from Integrated DNA Technologies (IDT). sgRNA activity against the region of interest was confirmed by using T7E1 cleavage assays, and then equimolar amounts of CRISPR RNA and trans-activating CRISPR RNA were mixed in IDT Duplex Buffer at final concentration of 10 μM and annealed at 95°C, followed by stepwise cooling (–5°C/min) to ambient temperature. This sgRNA complex was mixed with *Cas9* mRNA. For Z-allele insertion, the donor oligo was included and microinjected into ferret zygotes as described below.

For the generation of AAT-KO ferrets, exon 2 of the ferret *SERPINA1* gene was targeted using 2 sgRNA sequences: sgRNA E2-1 (CCTGGCCGACTTCGCCTTCAGCA) and sgRNA E2-2 (CGATGGCATCCTCCGTTCCCTGG).

For generation of the PiZZ-knockin ferrets, exon 5 of the ferret *SERPINA1* gene was targeted in the presence of a donor oligo using 2 sgRNA sequences: sgRNA E5-1 (TTGACGAGAAAGGGACAGAA) and sgRNA E5-2 (GAAAGGGACAGAAGCTACCG).

The donor oligo sequence was ACGGCCCCACTCAGAACAGGCCGGTGTCCTCTCTCCCTGCAGGGGGTGCATAAGGCTGTGCTGACCATTGACGAGAAAGGGACAGAAGCTACCGGGGCCACGTTTATGGAAGCCATCCCCATGTCGATGCCCCCAAGTGTCGA.

### Ferret zygote manipulation and cloning

The manipulation of ferret embryos is described in published reports (58, 68). For details, see Supplemental Methods.

### Genotyping

Genomic DNA was isolated from tail or ear snips using proteinase K digestion and phenol chloroform extraction (69). The region of interest was amplified by PCR (Supplemental Table 12). Products were resolved on a 1.0% agarose gel, then purified for Sanger sequencing (gel extraction kit, QIAGEN). Some amplicons, particularly from founders, were subcloned using topoisomerase-based cloning (TOPO cloning) and inserted into pCR2.1-TOPO-TA clone plasmids prior to sequencing (Invitrogen) using universal M13 forward/reverse primers (DNA Core Sequencing Facility at University of Iowa, Iowa Institute of Human Genetics, Genomics Division).

### NE inhibition assay

#### Plasma.

The antiprotease capacity of ferret plasma was determined using an NE activity assay protocol, as described by R&D Systems and in previous work (24). Briefly, plasma obtained by blood draw was centrifuged in plasma separation tubes (Sarstedt) and diluted 1:1000 in normal saline. Then, 0–5 μL of sample was added to 40 μL of 2 nM activated recombinant human NE (rhNE) in assay buffer (50 mM Tris, 1 M NaCl, 0.05% w/v Tween 20, pH 7.5) and brought to a final volume of 50 μL with assay buffer. The reaction was initiated by adding 50 μL of a 200 μM fluorogenic peptide substrate Gly-Arg-AMC (Bachem), and the fluorescence was measured (λ_ex_: 380 nm, λ_em_: 460 nm) using a SpectraMax i3x (Molecular Devices) in kinetic mode. A standard curve (*r*^2^ > 0.99) was generated by activating rhNE with recombinant mouse active cathepsin C (R&D Systems) and incubated at 37°C for 2 hours in activation buffer: 50 mM 2-(N-morpholino)ethanesulfonic acid, 50 mM NaCl, 5 mM DTT, pH 5.5.

#### BAL.

BAL from ferrets (0, 1, 3, 5, 7, and 10 μL) was added to 20 μg activated rhNE in assay buffer and brought to a volume of 50 μL. The reaction was initiated by the addition of 50 μL of a 200 μM fluorogenic peptide substrate (MEOSUC-Ala-Ala-Pro-Val-AMC) in assay buffer. Fluorescence was measured (λ_ex_: 380 nm, λ_em_: 460 nm) using a SpectraMax i3x (Molecular Devices) in kinetic mode for 5 minutes and normalized to the positive control well where no BAL (0 μL) was added. Given the slight changes in reagents and conditions from the plasma NE inhibition assay above, this system was validated by running plasma and similar results were obtained.

### Ferret anesthesia

Animals were anesthetized for pulmonary function tests (PFTs), QCT imaging, bronchoscopy, and LPS challenge by subcutaneous injection of ketamine (5–25 mg/kg) and xylazine (0.25–5 mg/kg). For longer experiments anesthesia was maintained with inhaled isoflurane (0%–5%) in oxygen. Throughout the procedure, trained personnel monitored the temperature, pulse, oxygen saturation, and respiratory rate, as well as its general clinical condition. For some experiments, anesthesia was reversed by intramuscular atipamezole (100 μL).

### Bronchoscopy and processing of BAL

BAL was obtained by flexible fiberoptic bronchoscopy from pairs of AAT-KO ferrets and controls matched for age, sex, and size. When bronchoscopy was performed on the same day as another procedure, BAL was the last intervention so that lavage would not affect imaging or lung mechanics. The experiment was conducted as described previously (24, 25). For details, see Supplemental Methods.

### PFTs

Anesthesia was induced as above and ferrets were intubated with an appropriately sized, cuffed endotracheal tube (inner diameter 2.5 or 3.0 mm). Tube confirmation was by visualization of passage through the cords and examining pressure tracings on the flexiVent ventilator. Routine ventilation parameters were tidal volume of 10 mL/kg; positive end-expiratory pressure (PEEP) of 3cmH_2_O; and respiratory rate of 60/min. This resulted in a mild hyperventilation; together with the administration of 2%–3% isoflurane, this left the animals sufficiently passive subjects for experiments without the need for paralysis.

The flexiVent system (SCIREQ) used initially included the standard base platform and an FX6 module piston, which can displace 96 mL/stroke. As larger ferrets have lung volumes that exceed the cylinder displacement volume, a 2-step maneuver was designed so that TLC can be reached in ferrets where a single-cylinder stroke cannot reach an airway pressure of opening of 30cmH_2_O. This multistroke maneuver used a first deep inflation from PEEP (3cmH_2_O) to 15cmH_2_O, followed by brief ventilation with a PEEP of 15cmH_2_O, and then a second inflation from 15cmH_2_O to 30cmH_2_O. These values, 3cmH_2_O–15cmH_2_O and 15cmH_2_O–30cmH_2_O, were added together to estimate the IC in larger animals. In experiments performed on smaller ferrets where this maneuver could be directly compared to a single-stroke deep inflation, the multistroke maneuver overestimated by 5%; this was controlled for in our study design by matching controls based on age, sex, and size.

Midway through the project our flexiVent platform was expanded to include a custom-built negative pressure forced exhalation (NPFE) system. This system was engineered in close collaboration with the manufacturer (SCIREQ) and is capable of accommodating a domestic ferret. This equipment includes a sealed plethysmography chamber with pneumotachograph screen, a negative pressure 50-liter reservoir, and a negative pressure controller with valve assembly that is controlled by the base system (Supplemental Figure 3). To verify the safety of this system, all perturbations were validated in WT ferrets not enrolled in the study.

The custom-written flexiVent script accounted for the size of the animal and included all perturbations for each experiment. Perturbations included (a) deep inflation to measure IC; (b) SnapShot-60 to assess dynamic compliance (Crs) and elastance (Ers) along with resistance (Rrs); (c) QuickPrime-6 to measure Newtonian resistance (Rn), tissue damping (G), and elastance (H); (d) a PV-loop to calculate quasistatic compliance (Cst); and (e) the NPFE maneuver to collect expiratory flows and volumes.

For all perturbations, the coefficient of determination for fitting the mathematical model had to be greater than 0.9 to be included in the average. It should be noted that the QuickPrime-6 perturbation rarely met criteria for adequate fit to the mathematical model; thus, the data in this manuscript do not include these parameters: Rn, G, and H.

### QCT imaging

Imaging was performed using a SOMATOM Force dual-source CT scanner (Siemens Healthcare GmbH) (24). Ferrets were anesthetized as above, intubated, and placed on the flexiVent system in the prone position. As in the PFT procedure, isoflurane was increased to 3%, ferrets were hyperventilated with 15cmH_2_O PEEP for 5 minutes to aid in recruitment, and it was ensured that they were passive on the ventilator. Ventilator settings were pressure control ventilation with a peak inspiratory pressure of 25cmH_2_O and PEEP 15cmH_2_O, rate of 60/min, and oxygen at 1–3 L/min. A customized deep inflation perturbation was performed with a slow ramp to 25cmH_2_O and held at that airway pressure while the CT scan was acquired at full inspiration (TLC), and the ferret was verified to not have breathed spontaneously. Ferrets were allowed to recover on the ventilator until they were able to breathe spontaneously. Care was taken to ensure that the field of view and scan parameters, including kernel, remained constant over time. For the SOMATOM Force, dose modulation was on with reference mAs of 40 and kVp of 70; slice thickness: 0.6 mm; slice interval: 0.3 mm; reconstruction kernel: Qr49; scan pitch: 1.2; rotation time: 0.5 seconds.

Quantification was performed by Pulmonary Analysis Software Suite, a software package developed in-house (70) that was heavily customized for use in ferret models of lung disease. In brief, a thresholding method is used, and the lung region and the outside airway structures are segmented based on the low pixel intensity. Then, airway structures are identified by region-growing methods and removed from the mask image, leaving only the lung region as marked (71, 72). Next, mediastinal areas are smoothed by 3D morphology close operation and holes inside the lung filled. Last, histogram measurements are carried out on the segmented lung region, and these values are grouped by HU thresholds for analysis and quantification.

### LPS challenge

See Supplemental Methods.

### Histopathology and alveolar morphometry

Ferrets were euthanized when in clinical distress or at the end of the study. The accessory (cordate) lobe of the lung was isolated. One half was embedded in optimal cutting temperature (OCT) medium for the preparation of frozen sections and the other snap-frozen in liquid nitrogen (LN_2_). All other lobes were inflated with 10% neutral buffered formalin (10% NBF) to a pressure of 25cmH_2_O and fixed in 10% NBF for more than 72 hours. The liver was fixed in 10% NBF, except for the caudate lobe, of which half was embedded in OCT and the other half in LN_2_. Other organs and tissues were fixed in 10% NBF and embedded in paraffin.

Lung sections (4 μm) were stained with H&E or diastase-resistant periodic acid–Schiff. For alveolar morphometry, H&E slides were imaged on a Nikon Super COOLSCAN 9000 ED at 100× original magnification using a custom adapter. Images were converted into 16-bit black-and-white files, then processed by a trained investigator in MetaMorph software using the integrated morphometry analysis module (MetaMorph off-line ver. 7.8.0.0, Molecular Devices LLC). The researcher identified and removed airways by recognizing pseudostratified epithelial regions, then determined the SA/P ratio of all alveolar spaces (73). Averages were calculated per lobe.

### Immunofluorescence staining

Frozen sections of liver (10 μm) were air-dried for 30 minutes and postfixed in 4% paraformaldehyde for 10 minutes. Slides were permeabilized in 0.2% Triton X-100 in PBS for 15 minutes, blocked with 5% donkey serum for 60 minutes, probed with mouse AAT antibody (1:500 dilution, catalog GTX83666, Genetex) at 4°C overnight, and incubated with Alexa Fluor 488 donkey anti–mouse IgG (Jackson ImmunoResearch) at ambient temperature for 2 hours. Slides were mounted with Vectashield containing DAPI (Vector Laboratories) and imaged on a Zeiss LSM700 confocal microscope.

### Single-molecule fluorescence RNA in situ hybridization

Frozen sections of liver (10 μm) were used for single-molecule fluorescence RNA in situ hybridization–based determination of gene expression, as previously described (25, 74). A type 1 probe targeting the ferret *SERPINA1* mRNA was designed and synthesized by Thermo Fisher Scientific based on National Center for Biotechnology Information accession number XM_004754758. Sections were processed and in situ hybridization was performed using the ViewRNA ISH Tissue 2-plex assay kit per the manufacturer’s instructions (Affymetrix). Nuclei were counterstained with DAPI before imaging on a Zeiss LSM700 confocal microscope.

### BAL proteomics

Unprocessed BAL was used for quantitative proteomics in this study. Other details are as described in previous reports (24, 25). For details, see Supplemental Methods.

### Quantitative PCR

Total RNA was isolated from tissues using TRIzol (Invitrogen); quality was assured by using those with an RNA integrity number greater than 9.0. cDNA synthesis was performed using the SuperScript III First-strand Synthesis System per the manufacturer’s instructions (Invitrogen). Sequences of TaqMan probes and primers are included in Supplemental Table 12. Probes were labeled with FAM fluorescent dye (IDT), and GAPDH served as an internal control. Quantitative PCR was performed on a Bio-Rad CFX Connect Real-Time system with this protocol: 95°C for 10 minutes, then 40 cycles of 95°C for 15 seconds, and 60°C for 1 minute. Relative quantification of *SERPINA1* (AAT) was performed using the ΔΔCt method.

### Western blotting of ferret plasma and tissues for AAT

See Supplemental Methods.

### Tests of hepatic function

Liver markers in PiZZ ferrets and controls were assessed by loading blood or plasma into a VetScan Mammalian Liver Profile cartridge according to the manufacturer’s instructions (Mammalian Liver Pro, 500-1040).

### Statistics

Data analysis was performed using Prism 9 software (GraphPad Software). For all experiments, data are shown as mean ± SEM unless indicated otherwise. Nonrepeated measures from each biological replicate were evaluated using 2-sample 2-tailed Student’s *t* test when 2 groups were compared or 1-way ANOVA with a specific posttest (as indicated in the legend of the relevant figure) when more than 2 groups were compared. For data sets that consisted of repeated measures, a mixed effects model was fitted in R (version 3.3.3; http://www.R-project.org/) using the lmer function within the R package lme4. Proteomics data were analyzed using Scaffold Q+S version 4.7 with a 0% FDR. *P* values less than 0.05 were considered statistically significant.

### Study approval

This study was performed according to protocols approved by the IACUC of the University of Iowa and conforms to NIH standards.

## Author contributions

JFE, BHR, and CM conceived the study design. NH, XL, JG, EAH, CM, JFE, and BHR designed experiments. NH, XL, ARV, TIAE, JSG, JG, SRM, LJG, ML, NM, XS, BL, ZY, ZF, LQ, YY, and BHR performed the experiments and acquired the data. NH, XL, ARV, TIAE, JSG, JG, SRM, ASJ, WS, KNGC, EAH, KW, JFE, and BHR analyzed and interpreted the data. BHR and JFE supervised the research team with assistance from XL. BHR and JFE wrote the manuscript with contributions from NH, XL, and CM. All authors gave final approval of the version to be published.

## Supplementary Material

Supplemental data

Supplemental table 1

Supplemental table 2

Supplemental table 3

Supplemental table 4

Supplemental table 5

Supplemental table 6

Supplemental table 7

Supplemental table 8

Supplemental table 9

Supplemental table 10

Supplemental table 11

Supplemental table 12

## Version 1 02/01/2022

In-Press Preview

## Version 2 03/08/2022

Electronic publication

## References

[1] Lozano R (2012). Global and regional mortality from 235 causes of death for 20 age groups in 1990 and 2010: a systematic analysis for the Global Burden of Disease study 2010. Lancet.

[2] Buist AS (2007). International variation in the prevalence of COPD (the BOLD study): a population-based prevalence study. Lancet.

[3] Tuder RM (2010). Lung disease associated with alpha1-antitrypsin deficiency. Proc Am Thorac Soc.

[4] Crystal RG (1990). Alpha 1-antitrypsin deficiency, emphysema, and liver disease. Genetic basis and strategies for therapy. J Clin Invest.

[5] Stoller JK, Aboussouan LS (2005). Alpha1-antitrypsin deficiency. Lancet.

[6] Koopman P (1989). Widespread expression of human alpha 1-antitrypsin in transgenic mice revealed by in situ hybridization. Genes Dev.

[7] Carlson JA (1988). Multiple tissues express alpha 1-antitrypsin in transgenic mice and man. J Clin Invest.

[8] Travaglini KJ (2020). A molecular cell atlas of the human lung from single-cell RNA sequencing. Nature.

[9] Voynow JA (2008). Proteases and cystic fibrosis. Int J Biochem Cell Biol.

[10] Lomas DA (2016). Does protease-antiprotease imbalance explain chronic obstructive pulmonary disease?. Ann Am Thorac Soc.

[11] Curiel D (1989). Alpha 1-antitrypsin deficiency caused by the alpha 1-antitrypsin Nullmattawa gene. An insertion mutation rendering the alpha 1-antitrypsin gene incapable of producing alpha 1-antitrypsin. J Clin Invest.

[12] Lee JH, Brantly M (2000). Molecular mechanisms of alpha1-antitrypsin null alleles. Respir Med.

[13] Kelly E (2010). Alpha-1 antitrypsin deficiency. Respir Med.

[14] Gooptu B (2009). Mechanisms of emphysema in alpha1-antitrypsin deficiency: molecular and cellular insights. Eur Respir J.

[15] Rudnick DA, Perlmutter DH (2005). Alpha-1-antitrypsin deficiency: a new paradigm for hepatocellular carcinoma in genetic liver disease. Hepatology.

[16] Carlson JA (1989). Accumulation of PiZ alpha 1-antitrypsin causes liver damage in transgenic mice. J Clin Invest.

[17] Marcelino MY (2014). Animal models in chronic obstructive pulmonary disease-an overview. Exp Lung Res.

[18] Ni K (2016). Alpha-1 antitrypsin investigations using animal models of emphysema. Ann Am Thorac Soc.

[19] Barbour KW (2002). The murine alpha(1)-proteinase inhibitor gene family: polymorphism, chromosomal location, and structure. Genomics.

[20] Borel F (2018). Editing out five Serpina1 paralogs to create a mouse model of genetic emphysema. Proc Natl Acad Sci U S A.

[21] Kobayashi S (2013). A single dose of lipopolysaccharide into mice with emphysema mimics human chronic obstructive pulmonary disease exacerbation as assessed by micro-computed tomography. Am J Respir Cell Mol Biol.

[22] De Langhe E (2012). Quantification of lung fibrosis and emphysema in mice using automated micro-computed tomography. PLoS One.

[23] Raju SV (2016). A ferret model of COPD-related chronic bronchitis. JCI Insight.

[24] Rosen BH (2018). Infection is not required for mucoinflammatory lung disease in CFTR-knockout ferrets. Am J Respir Crit Care Med.

[25] Sun X (2019). In utero and postnatal VX-770 administration rescues multiorgan disease in a ferret model of cystic fibrosis. Sci Transl Med.

[26] Fernandez-Petty CM (2019). A glycopolymer improves vascoelasticity and mucociliary transport of abnormal cystic fibrosis mucus. JCI Insight.

[27] Swatek AM (2018). Depletion of airway submucosal glands and TP63^+^KRT5^+^ basal cells in obliterative bronchiolitis. Am J Respir Crit Care Med.

[28] Evans TIA (2016). Glandular proteome identifies antiprotease cystatin C as a critical modulator of airway hydration and clearance. Am J Respir Cell Mol Biol.

[29] Tanash HA (2008). Clinical course and prognosis of never-smokers with severe alpha-1-antitrypsin deficiency (PiZZ). Thorax.

[30] Ogushi F (1991). Risk factors for emphysema. Cigarette smoking is associated with a reduction in the association rate constant of lung alpha 1-antitrypsin for neutrophil elastase. J Clin Invest.

[31] Pellegrino R (2005). Interpretative strategies for lung function tests. Eur Respir J.

[32] Agusti A, Hogg JC (2019). Update on the pathogenesis of chronic obstructive pulmonary disease. N Engl J Med.

[33] Celli BR, Wedzicha JA (2019). Update on clinical aspects of chronic obstructive pulmonary disease. N Engl J Med.

[34] Bhatt SP (2019). Discriminative accuracy of FEV1:FVC thresholds for COPD-related hospitalization and mortality. JAMA.

[35] Gappa M (2001). Lung function testing in infants with cystic fibrosis: lessons from the past and future directions. Pediatr Pulmonol.

[36] Linnane BM (2008). Lung function in infants with cystic fibrosis diagnosed by newborn screening. Am J Respir Crit Care Med.

[37] Gevenois PA (1996). Comparison of computed density and microscopic morphometry in pulmonary emphysema. Am J Respir Crit Care Med.

[38] Eda S (1997). The relations between expiratory chest CT using helical CT and pulmonary function tests in emphysema. Am J Respir Crit Care Med.

[39] Kubo K (1999). Expiratory and inspiratory chest computed tomography and pulmonary function tests in cigarette smokers. Eur Respir J.

[40] Schroeder JD (2013). Relationships between airflow obstruction and quantitative CT measurements of emphysema, air trapping, and airways in subjects with and without chronic obstructive pulmonary disease. AJR Am J Roentgenol.

[41] Tu C (2014). Large-scale, ion-current-based proteomics investigation of bronchoalveolar lavage fluid in chronic obstructive pulmonary disease patients. J Proteome Res.

[42] Ohlmeier S (2016). Lung tissue proteomics identifies elevated transglutaminase 2 levels in stable chronic obstructive pulmonary disease. Am J Physiol Lung Cell Mol Physiol.

[43] Stites SW (1995). Transferrin concentrations in serum and lower respiratory tract fluid of mechanically ventilated patients with COPD or ARDS. Chest.

[44] Chen WQ (2018). Influences of PON1 on airway inflammation and remodeling in bronchial asthma. J Cell Biochem.

[45] Stoltz DA (2008). Drosophila are protected from Pseudomonas aeruginosa lethality by transgenic expression of paraoxonase-1. J Clin Invest.

[46] Mayer AS (2006). Risk factors for symptom onset in PI*Z alpha-1 antitrypsin deficiency. Int J Chron Obstruct Pulmon Dis.

[47] Faull SV (2020). The structural basis for Z α_1_-antitrypsin polymerization in the liver. Sci Adv.

[48] Piitulainen E (1997). Effect of age and occupational exposure to airway irritants on lung function in non-smoking individuals with alpha 1-antitrypsin deficiency (PiZZ). Thorax.

[49] Fuchs SI (2016). Lung clearance index for monitoring early lung disease in alpha-1-antitrypsin deficiency. Respir Med.

[50] Demeo DL (2007). Determinants of airflow obstruction in severe alpha-1-antitrypsin deficiency. Thorax.

[51] Bergin DA (2014). The circulating proteinase inhibitor α-1 antitrypsin regulates neutrophil degranulation and autoimmunity. Sci Transl Med.

[52] Bergin DA (2010). α-1 Antitrypsin regulates human neutrophil chemotaxis induced by soluble immune complexes and IL-8. J Clin Invest.

[53] Geraghty P (2014). α1-Antitrypsin activates protein phosphatase 2A to counter lung inflammatory responses. Am J Respir Crit Care Med.

[54] Jonigk D (2013). Anti-inflammatory and immunomodulatory properties of α1-antitrypsin without inhibition of elastase. Proc Natl Acad Sci U S A.

[55] Kotke V (2017). [Alpha-2 macroglobulin serum level in patients with alpha-1 antitrypsin deficiency]. Pneumologie.

[56] Wewers MD (1988). Alveolar fluid neutrophil elastase activity in the adult respiratory distress syndrome is complexed to alpha-2-macroglobulin. J Clin Invest.

[57] Sveger T (1998). Adolescents with alpha1-antitrypsin deficiency have high alpha2-macroglobulin and low neutrophil lipocalin and elastase levels in plasma. Pediatr Res.

[58] Yu M (2019). Highly efficient transgenesis in ferrets using CRISPR/Cas9-mediated homology-independent insertion at the ROSA26 locus. Sci Rep.

[59] Peng X (2014). The draft genome sequence of the ferret (mustela putorius furo) facilitates study of human respiratory disease. Nat Biotechnol.

[60] Molloy K (2014). Clarification of the risk of chronic obstructive pulmonary disease in α1-antitrypsin deficiency PiMZ heterozygotes. Am J Respir Crit Care Med.

[61] Strnad P (2020). Alpha1-antitrypsin deficiency. N Engl J Med.

[62] Parr DG (2004). Pattern of emphysema distribution in alpha1-antitrypsin deficiency influences lung function impairment. Am J Respir Crit Care Med.

[63] Fregonese L (2008). Alpha-1 antitrypsin Null mutations and severity of emphysema. Respir Med.

[64] Brantly ML (1991). Use of a highly purified alpha 1-antitrypsin standard to establish ranges for the common normal and deficient alpha 1-antitrypsin phenotypes. Chest.

[65] Bornhorst JA (2013). α1-antitrypsin phenotypes and associated serum protein concentrations in a large clinical population. Chest.

[66] Donato LJ (2012). Reference and interpretive ranges for α(1)-antitrypsin quantitation by phenotype in adult and pediatric populations. Am J Clin Pathol.

[67] Sveger T (1976). Liver disease in alpha1-antitrypsin deficiency detected by screening of 200,000 infants. N Engl J Med.

[68] Li Z (2006). Cloned ferrets produced by somatic cell nuclear transfer. Dev Biol.

[69] Strauss WM (2001). Preparation of genomic DNA from mammalian tissue. Curr Protoc Mol Biol.

[70] http://www.lungworkshop.org/2009/proceedings-2008.html.

[71] Tschirren J (2005). Intrathoracic airway trees: segmentation and airway morphology analysis from low-dose CT scans. IEEE Trans Med Imaging.

[72] Tschirren J (2005). Matching and anatomical labeling of human airway tree. IEEE Trans Med Imaging.

[73] Parameswaran H (2006). Quantitative characterization of airspace enlargement in emphysema. J Appl Physiol (1985).

[74] Yi Y (2016). A transient metabolic recovery from early life glucose intolerance in cystic fibrosis ferrets occurs during pancreatic remodeling. Endocrinology.

