# Supplementary material for: Ferret models of alpha-1 antitrypsin deficiency develop lung and liver disease
Source: JCI Insight. 2022 Mar 8;7(5):e143004. doi: 10.1172/jci.insight.143004 (PMC8983124; doi:10.1172/jci.insight.143004)
Supplement: Supplemental data [file jciinsight-7-143004-s036.pdf]

Supplemental Methods:

Ferret zygote manipulation and cloning. The manipulation of ferret embryos is described in published reports (1, 2). Briefly, 7-month-old wild-type (WT) sable or albino coat-color virgin jills (in estrus) were bred to WT hobbs of the same coat-color, and fertilized single-cell embryos (zygotes) were collected 46-48 hours after mating. Collected zygotes were incubated in pre-warmed M199 medium for 2-4 hours and then transferred, in MPBS (2 grams BSA in 500ml PBS) buffer, for microinjection using an Eppendorf microinjection system (Femtojet, Eppendorf AG, Hamburg, Germany). The mixture of gRNA or gRNA-donor oligo (25ng/µl each) and Cas9 mRNA (100ng/µl) was continuously injected into the cytosol of zygotes using an Eppendorf microinjection needle (Femtotips II, Eppendorf). The injected zygotes were then transferred into M199 medium with 10% fetal calf serum (FCS) and cultured at 38.5°C overnight. Zygotes that developed into the 2-cell or 4-cell stage were selected and transplanted into primipara surrogate recipient jills with a different coat-color than that of the donor zygotes. After 42-days of gestation (full-term), the kits were delivered naturally. Genotype was determined as below and heterozygous founders were crossed to generate homozygous AAT-deficient animals (AAT-KO or PiZZ) for study. Controls were age-, gender-, and size matched WT ferrets and were kept in the same facilities to help control for exposures (Supplemental Figure 1; Supplemental Tables 1, 2, 10 and 11).

BAL proteomics. Unprocessed BAL was used for quantitative proteomics in this study. Other details are as described in previous reports (3, 4).

BAL sample preparation, digestion, labeling, and loading on columns. Briefly, 10µg protein was aliquoted into MS-grade Eppendorf tubes and diluted to 50µl using 8M urea. Proteins were reduced in 0.1M dithiothreitol (DTT) at 37°C for 1 hour, and then alkylated using 0.55M iodoacetamide at ambient temperature for 1 hour in the dark. Samples were brought to a final urea concentration of 1M by dilution with 0.1M triethylammonium bicarbonate (TEAB, pH 8.0; final volume 400µl), and digested overnight with trypsin/Lys C (1:50 enzyme ratio). Digested samples were subjected to “light” and “heavy” stable isotope dimethyl labeling where the control samples were treated with 16µl of 4% (v/v) light formaldehyde (CH_2O) and AAT-

KO samples were treated with 16µl of 4% (v/v) heavy formaldehyde ($^{13}CD_2O$). Each labelled sample received another 16µl of freshly prepared 0.6M cyanoborohydrade ($NaBH_3CN$ and $NaBD_3CN$ for the light and heavy labeled samples, respectively). The samples were incubated for 2 hours at ambient temperature on a shaker, then labeling was terminated with 16µl of 1% (v/v) NH_4OH in H_2O. Reaction mixtures were acidified using 5% (v/v) formic acid to further quench the reaction. Labeled pairs were then mixed thoroughly at a ratio of 1:1, desalted on a C_{18}-micro spin column (The Nest Group, Inc; Southborough, MA, USA), and applied to a SCX-micro spin column (The Nest Group, Inc). They were then eluted using 5 concentrations of salt buffers (15, 20, 30, 60, and 120mM KCl) to decrease the complexity of peptides in the combined samples. These 5 fractions were desalted and 2µl of each sample was loaded onto a home-packed C-18 column (Halo 2.7µm particles: MDC) 100µm i.d. × 10cm using the Thermo EZ nLC 1200. The data were analyzed using a shotgun protocol on an Orbitrap Fusion Lumos MS (ThermoFisher Scientific, San Jose, CA, USA) coupled to a Thermo Easy-nLC1200 set to support an Integra Frit sample trap (New Objective, 75µm i.d. × 2.5cm) containing an Upchurch Sample Trap holder (part No. C-1600 from IDEX). The analytic column was a self-packed 360µm o.d. × 100µm i.d. fused silica column pulled to an emitter diameter of ~5-6µm. It was packed with 10cm of Halo 2.7µm solid core C-18 particles. Peptides were separated in-line by the MS, using a 100-minute gradient composed of linear and static segments wherein Buffer A is 0.1% formic acid and Buffer B is 80% acetonitrile with 0.1% formic acid. The gradient began at 3% for 3 minutes and then transitioned as follows (%B, min): (7,2), (26,62), (44,78), (44,86), (98,91), (98,100).

Tandem mass spectrometry on the LUMOS Orbitrap. The scanning of sequences began with a full survey (m/z 350-1500) acquired on an Orbitrap Fusion Lumos mass spectrometer (Thermo Fisher Scientific) at a resolution of 60,000 in the off axis Orbitrap segment (MS1). MS1 scans were acquired at 3-second intervals during the 120-minute gradient described above. The most abundant precursors were selected from among the 2-8 charge state ions at a $2.0x10^5$ threshold. Ions were dynamically excluded for 30 seconds if they were targeted twice during the prior 30-second period. Selected ions were isolated by a multi-segment quadrupole with a mass window on m/z 2, then sequentially subjected to collision-induced dissociation (CID) and high-energy collision-induced dissociation (HCD) activation conditions, in the ion trap and the ion routing multipole, respectively. The automatic gain control target (AGC) for CID was $4.0x10^4$, 35% collision energy, an

activation Q of 0.25 and a 100 milliseconds maximum fill time. Targeted precursors were also fragmented by HCD at 40% collision energy, and an activation Q of 0.25. HCD fragment ions were analyzed using the Orbitrap (AGC 1.2x10^5, maximum injection time 110ms, and resolution set to 30,000 at 400 Th). Both MS2 channels were recorded as centroid, and the MS1 survey scans were recorded in profile mode.

Proteomic searches. Initial spectral searches were performed using Mascot Server (ver. 2.6), with quantification for the dimethyl labels (+28 and +36) performed by Mascot Distiller (ver. 2.6). Searches were performed against the NCBInr database for ferret (9669) downloaded on February 20, 2017; 48,113 sequences were searched. Searches were also conducted against an equal number of decoy entries, which were created by reversing the original entries in the 9669 database. For both searches, discriminant scores were determined by Scaffold Q+S ver. 4.7 (Proteome Software, Portland, OR, USA) at 0% FDR, and statistical testing was accomplished using the algorithms accompanying Scaffold. The databases used included the recent ferret mucin annotations for proteomics developed by Dr. Gunnar Hansson for MUC1, 2, 5ac, 5b, 6, 7, 16, and 19 (http://www.medkem.gu.se/mucinbiology/databases/db/Mucin-ferret-2015.htm). Additional analysis was conducted using the Ingenuity Pathway Analysis (IPA) database and software (Ingenuity Systems, Redwood City, CA, USA), leaving out *SERPINA1* so that this data point would not skew the analysis.

Lipopolysaccharide (LPS) challenge. Two-month-old ferret kits underwent intratracheal administration of LPS (2mg/kg LPS in 2ml saline, serotype 055:B5 Escherichia coli LPS, L2880; Sigma-Aldrich). This was followed by a second dose at half the concentration (1mg/kg in 2ml saline) two weeks later. After each challenge, the ferrets were monitored clinically to ensure safety and tolerability. PFTs were performed on the flexiVent system as described, and the animals were sacrificed at approximately 8-months-of-age for alveolar morphometry.

Bronchoscopy and processing of bronchoalveolar lavage (BAL). BAL was obtained by flexible fiberoptic bronchoscopy from pairs of AAT-KO ferrets and controls matched for age, sex, and size. When bronchoscopy

was performed on the same day as another procedure, BAL was the last intervention so that lavage would not affect imaging or lung mechanics. The experiment was conducted as described previously (3, 4).

Bronchoscopy. In brief, ferrets were anesthetized as above and maintained with isoflurane 0-5% in oxygen. The bronchoscope (Pentax FB-8V; Pentax Medical, Akishima, Tokyo, Japan) was inserted orally and the suction channel was not used until the bronchoscope tip was wedged in the airway of interest. Two aliquots (1.5ml/kg body weight each; total volume of 3ml/kg) of sterile 0.9% saline (NaCl) were instilled and then recovered with gentle manual aspiration.

BAL processing. BAL aliquots were pooled and then divided into two portions. One was snap frozen in liquid nitrogen (LN_2) without processing and stored at -80°C, for subsequent use in proteomics analyses. The second portion was centrifuged (500g for 5 minutes at 4°C), after which the supernatant was removed and snap frozen in aliquots. The BAL pellet was resuspended and snap frozen in LN_2. For some experiments, a portion of the resuspended BAL pellet was spun onto slides using a Cytospin 3 at 800rpm for 3 minutes after RBC lysis (BD PharmLyse, San Diego, CA USA). BAL cell slides were stained with a Diff-Qwik Stain kit (Siemens, Newark, DE USA) and a blinded investigator counted cell types as previously described (3) and per established guidelines (5).

Western blotting for ferret plasma AAT. Plasma was diluted 1:20 in sterile saline (NaCl) and then mixed with 6× Laemli buffer and denatured at 95°C for 5 minutes. Ferret BAL was concentrated using Centricon filters with a 3,000 Dalton cutoff, 20μg of protein was mixed with 6× Laemli buffer and denatured as above. An equal amount of protein in 18μl was loaded and resolved by SDS-PAGE, then transferred on to a nitrocellulose membrane (Supplemental Figure 9). Ponceau S stain (0.1%, Sigma, St Louis, MO, USA) was used to view proteins and captured as a loading control. The membrane was rinsed in ddH_2O and blocked in 4% non-fat milk in PBST (0.1% Tween-20 in PBS) for 1 hour at ambient temperature. The blocked membrane was probed with rabbit anti-AAT antibody (1:5,000 in blocking buffer; OriGene TA321103, Rockville, MD, USA) overnight at 4°C, washed with PBST three times, incubated with IR-dye 800CW donkey

anti-rabbit IgG (1:10,000 in blocking buffer; 926-32213, Li-Cor Biosciences, Lincoln, NE, USA) for 1 hour at ambient temperature, washed, and imaged on a Li-Cor Odyssey scanner (Li-Cor Biosciences).

Immunoblotting for ferret liver tissues. Tissues were homogenized in native lysis buffer (20mM Tris pH 7.4, 150mM NaCl, 1% v/v NP40, 10mM N-ethylmaleimide [NEM], and Roche protease inhibitor cocktail), centrifuged for 10 minutes at 9,600×g, and the soluble supernatant was removed for subsequent analysis. For native (non-denaturing) polyacrylamide gel electrophoresis (native PAGE), soluble proteins were diluted in native lysis buffer to a final concentration of 5µg/µl along with 10% glycerol and 0.01% bromophenol blue. For SDS-PAGE, Laemmli buffer was used to dilute soluble proteins to a final concentration of 5µg/µl, heat denatured at 95°C for 5 minutes, allowed to cool, and then loaded. Ferret plasma was processed as above, diluted 20-fold in 0.9% saline, then 2× Laemmli buffer added, heat denatured at 95°C for 5 minutes, allowed to cool, and finally loaded. 50µg of native or denatured protein samples (10µl volume at 5µg/µl) were loaded and resolved by 10% native PAGE or SDS-PAGE, as indicated. The separated proteins were transferred to PVDF membrane over 60 minutes using a constant current of 400mA. Membranes were blocked with non-fat milk (4% in TBST; 0.2% Tween-20) for 1 hour at ambient temperature and washed with TBST. Blocked membranes were probed with the following primary antibodies in blocking buffer (1:5,000 in TBST with 4% non-fat milk) overnight at 4°C: rabbit anti-AAT antibody (OriGene TA321103, Rockville, MD, USA); goat anti-GAPDH (ThermoFisher, PA1-9046); or sheep anti-albumin (Novus Biologicals, NB120-8940). Probed membranes were washed in blocking buffer three times (15 minutes/wash), before incubating with secondary antibodies (1:10,000 in blocking buffer) for 1 hour at ambient temperature. The blots were washed three times in blocking buffer (10 minutes/wash), followed by washing three times in TBST (5 minutes/wash), before the protein of interest was visualized using a Li-Cor Odyssey scanner (Li-Cor Biosciences, Lincoln, NE, USA).

Supplemental Data:

Supplemental Figure 1

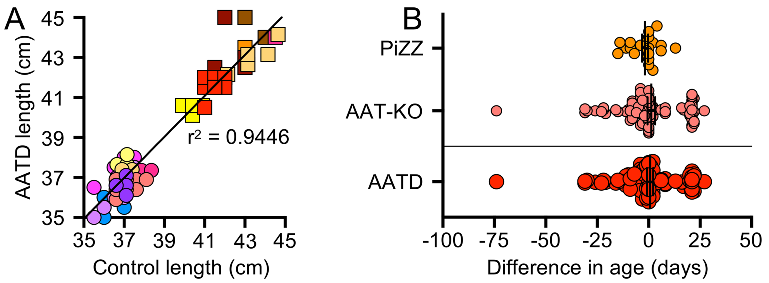

Supplemental Figure 1. Comparison of ferret demographics and matching for PFT measurements. (**A**) Comparison of body length for each AATD (AAT-KO or PiZZ) ferret to that of its matched control. Each data point represents one measurement of a fully-grown ferret; males are represented by squares and females by circles. Colors denote separate pairs of animals (i.e, AAT-KO or PiZZ and its control). Note that the points are slightly offset to better display overlapping data. (**B**) Difference in age at the time of flexiVent PFT experiments for AAT-KO (light red) and PiZZ (orange) ferrets and its respective control, with both genotypes combined in AATD (red). Each data point represents one measurement of age for (AATD – control). Data are represented as mean±SEM.

Supplemental Figure 2

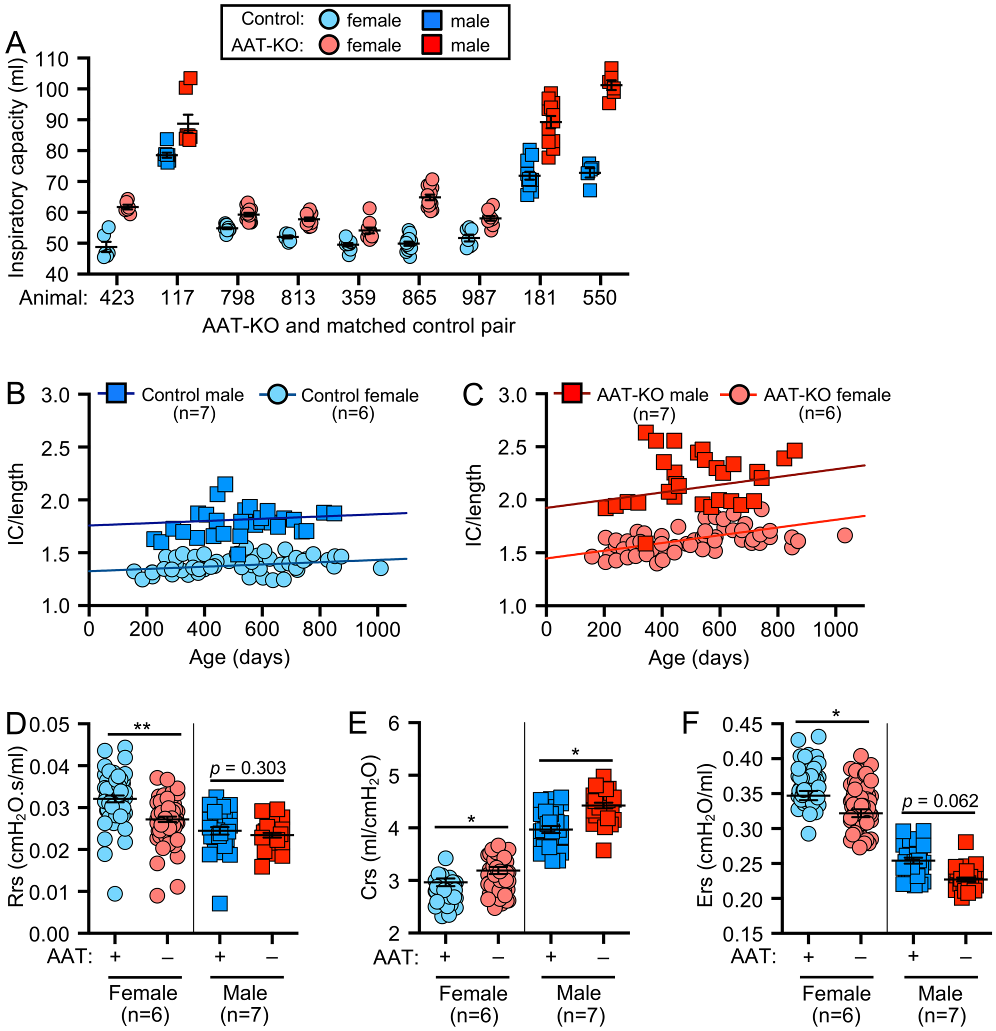

Supplemental Figure 2. Absence of alpha-1 antitrypsin (AAT) alters inspiratory capacity, pulmonary resistance, dynamic compliance, and elastance. (**A**) Inspiratory capacity (IC, ml) at 30 cmH_2O in matched pairs of AAT-KO and control ferrets in which multiple measurements were obtained during the study (n=13 pairs in the study including single measurements; *P*-value by mixed effects model, *p*=0.0004). (**B** and **C**) IC normalized to body length (IC/length) is shown over time for control (B) and AAT-KO (C) ferrets where each data point is one measurement. A mixed effect model was fit to each genotype and was the same for separate sexes, as the interaction term for sex was not significant (*p*=0.1396). The slope for control male and female ferrets in (B) is 0.00011 and for AAT-KO male and female ferrets in (C) is 0.00036. (**D**) Resistance of the respiratory system (Rrs, $cmH_2O.s/ml$) in matched pairs of AAT-KO and control ferrets, from which multiple measurements were obtained during the study. Individual measurements are represented by data points and plotted per group (n=29-63 experiments in 13 paired animals; *P*-value by mixed effects model in, *p*=0.0062 for females and *p*=0.303 for males). (**E**) Dynamic compliance of the respiratory system (Crs, ml/cmH_2O) in matched pairs depicted in groups (n=29-64 experiments in 13 paired animals; *P*-value by mixed effects model, *p*=0.037 for females and *p*=0.042 for males). (**F**) Elastance of the respiratory system (Ers, cmH_2O/ml) in matched pairs depicted in groups (n=29-64 experiments in 13 paired animals; *P*-value by mixed effects model, *p*=0.025 in females and *p*=0.062 in males). In all panels, squares indicate male ferrets and circles female. Panels show mean±SEM. **p*<0.05; ***p*<0.01

Supplemental Figure 3

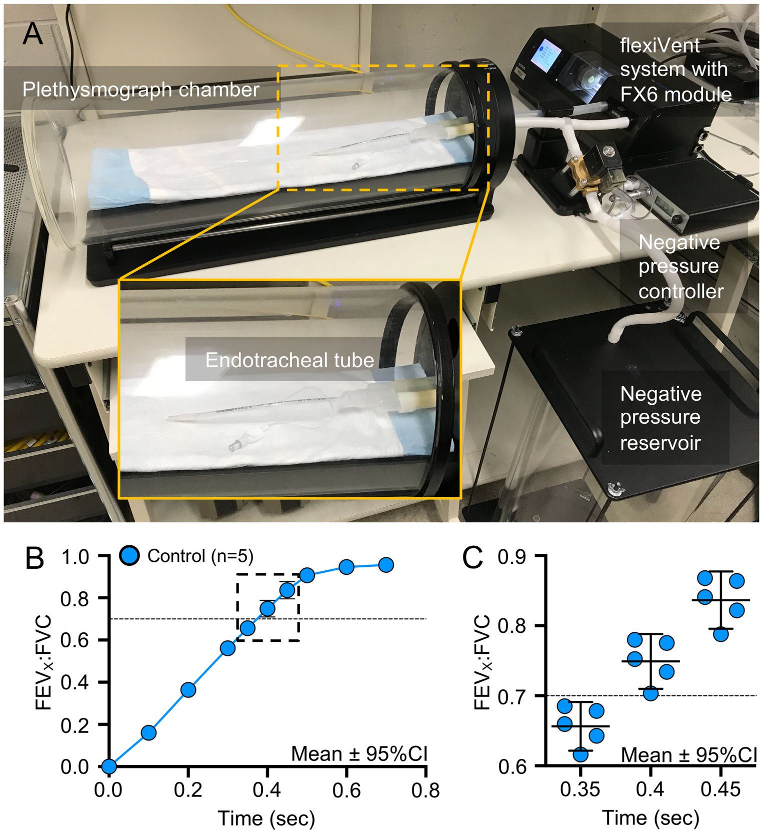

Supplemental Figure 3. **Custom-designed flexiVent negative pressure forced exhalation (NPFE) module for ferret PFTs and determination of FEV$_X$ in wild-type (WT) ferrets**. (**A**) Photograph of SCIREQ flexiVent system. The FX6 module is installed and the custom-designed NPFE extension that includes body plethysmograph chamber with installed endotracheal tube, negative pressure controller, and reservoir are attached. (**B**) Ratio of forced expiratory volume at each timepoint (FEV$_X$) to forced vital capacity (FVC) in a cohort of WT ferrets (n=5 WT ferrets). The boxed region is expanded in (C). (**C**) Ratio of FEV$_X$:FVC at the three most promising timepoints (0.35, 0.4, and 0.45 seconds) shown in relation to threshold ratio of 0.7. Data are shown as mean±95% confidence interval (95%CI); some error bars are hidden by symbols.

Supplemental Figure 4

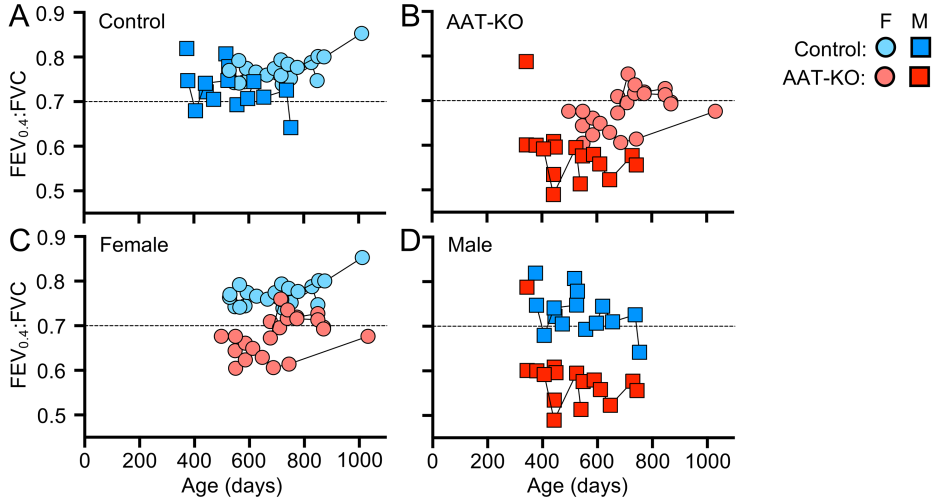

Supplemental Figure 4. **$FEV_{0.4}$:FVC ratio over time for AAT-KO ferrets and matched controls**. (**A**–**D**) Ratio of $FEV_{0.4}$:FVC as a function of days, for AAT-KO ferrets and age-/size-matched controls. The group is shown broken down by genotype, with controls in blue (A) and AAT-KO in red (B), as well as by sex with females depicted with circles (C) and males with squares (D). In all panels, lines connect measurements in the same ferret over time (n=35-38 measurements in eleven pairs of matched animals; *P*-value by mixed effects model was not significant when considering age).

Supplemental Figure 5

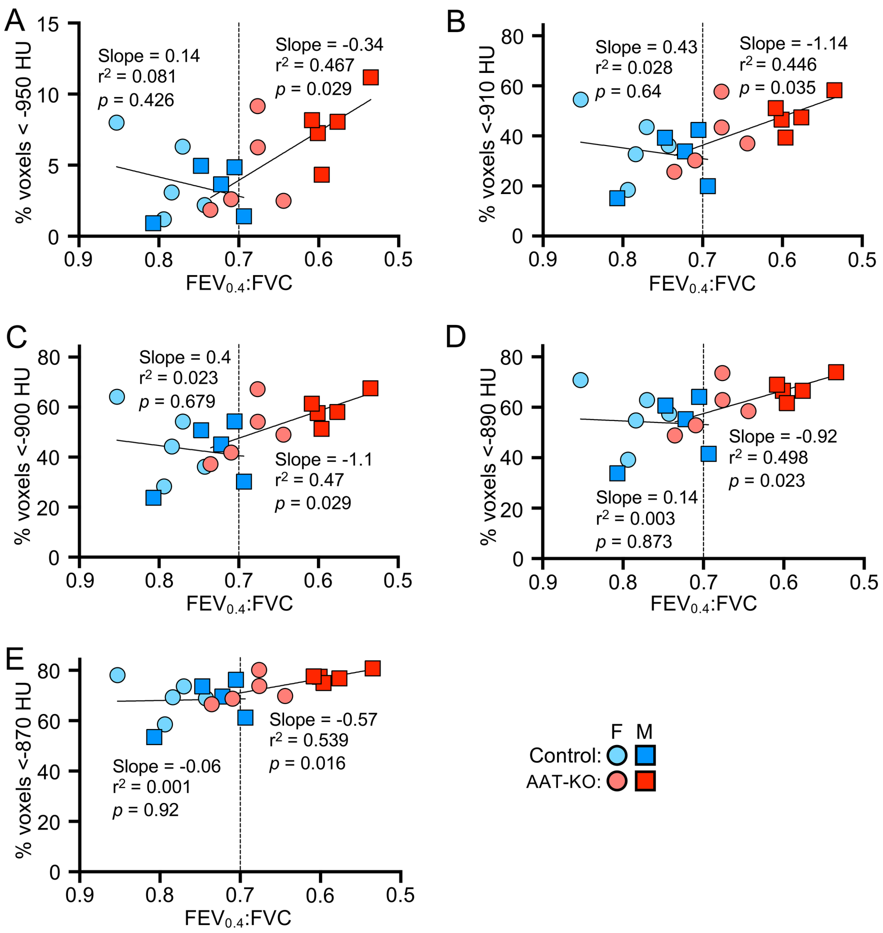

Supplemental Figure 5. **Emphysema drives airflow obstruction in alpha-1 antitrypsin knockout (AAT-KO) ferrets**. (**A**–**E**) Quantitative computed tomography (QCT) analysis of AAT-KO and control ferrets at total lung capacity (TLC, airway pressure=25cmH_2O) with indicated standard Houndsfield Unit (HU) thresholds plotted against the $FEV_{0.4}$:FVC ratio for each ferret. Linear regression model was fit to the data with the indicated parameters on each side for respective genotypes (n=10 matched ferret pairs; *P*-value by linear regression model parameters as indicated for each genotype). In all panels, squares represent males and circles represent females.

Supplemental Figure 6

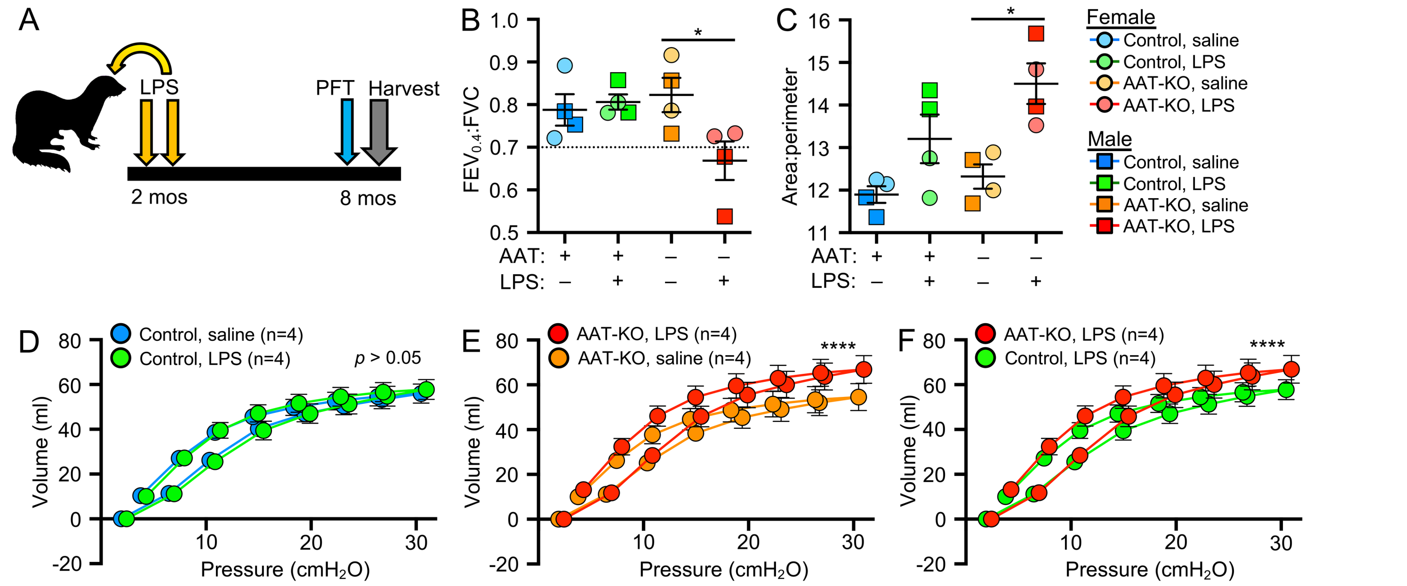

Supplemental Figure 6. **Inflammatory insults lead to increased severity of obstructive physiology and airspace enlargement**. (**A**) Timeline for LPS challenge that was delivered as a first dose at 2-months-old, with a second half-dose two weeks after the first. Pulmonary function tests (PFTs) were performed at about 6-months-old and ferrets were sacrificed at 8-months-old. (**B**) $FEV_{0.4}$:FVC ratio for the indicated groups (n=4 per group; *P*-value by one-way ANOVA and Tukey's post-test, *p=0.049). (**C**) Morphologic quantification of airspace enlargement (n=4 per group; *P*-value by one-way ANOVA and Tukey's post-test, *p=0.0126). (**D–F**) Pressure-volume loops (PV-loops) comparing various treatment groups: (D) controls exposed to both saline and LPS; (E) AAT-KO exposed to saline and LPS; and (F) control and AAT-KO receiving LPS (n=4 for each group; *P*-value by quadratic regression; ****p<0.0001). All graphs show mean±SEM; some error bars are hidden by symbols. In B-C, squares indicate males while females are lighter colored circles. Data are compared as indicated within the description for each graph above. *p<0.05; ****p<0.0001.

Supplemental Figure 7

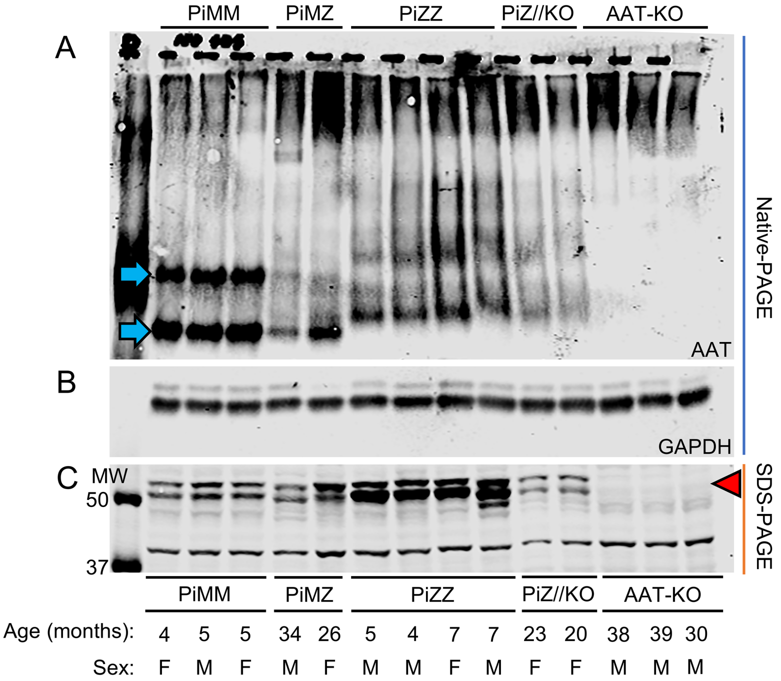

Supplemental Figure 7. PiZ-AAT polymerization in liver tissue from PiZZ, PiMZ, and PiZ//KO ferrets. (**A**) Native PAGE blot probed with anti-AAT antibody showing altered migration pattern in PiZZ, PiMZ, and PiZ//KO ferret liver tissues compared with PiMM controls (blue arrows indicated PiM band). AAT-KO samples are at the far right. (**B**) Native PAGE blot probed for GAPDH as loading control for the samples. (**C**) SDS-PAGE blot probed with anti-AAT antibody as comparison to (A). Arrowhead in red marks the expected size of AAT, slightly larger than 50kDa. Molecular weight markers are in the left-most lane. Ages of each ferret at harvest, in months, along with the sex are listed along the bottom of the figure.

Supplemental Figure 8

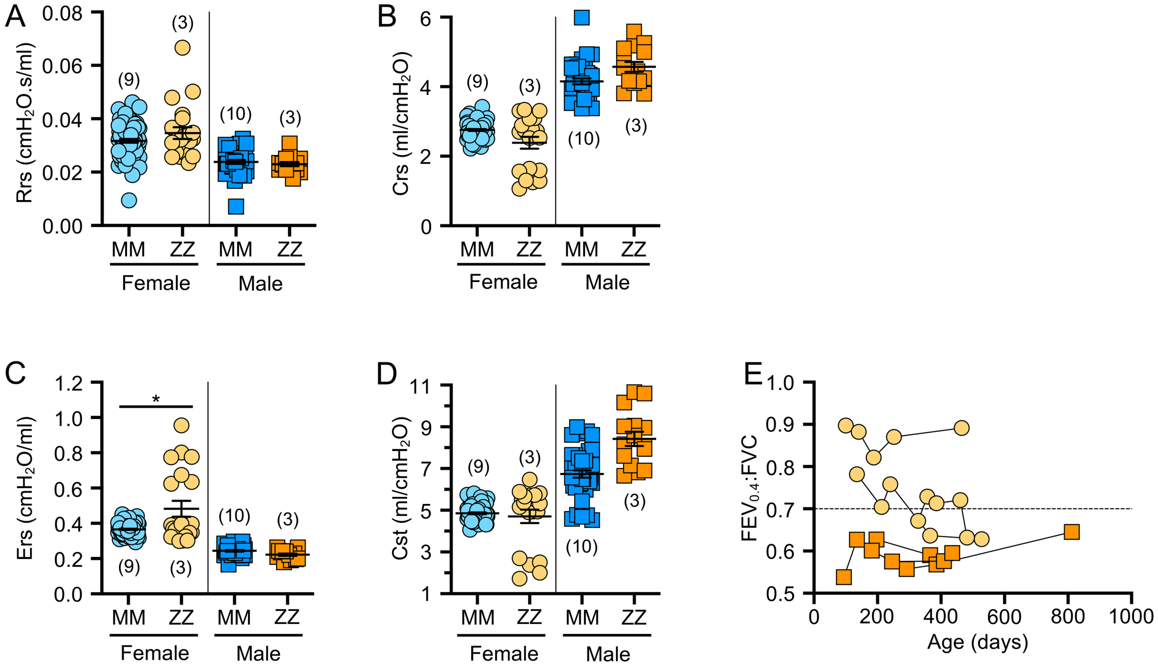

Supplemental Figure 8. PiZZ ferret pulmonary resistance, compliance, elastance, and $FEV_{0.4}$:FVC ratio. (**A**) Resistance of the respiratory system (Rrs, cmH_2O.s/ml) in PiZZ and PiMM control ferrets in which multiple measurements were obtained during the study. Individual measurements are represented by data points graphed separately by gender (n=36-118 experiments in 6 PiZZ and 19 PiMM control animals; *P*-value by mixed effects model, *p*=0.379 for females and *p*=0.652 for males). (**B**) Dynamic compliance of the respiratory system (Crs, ml/cmH_2O) in PiZZ and PiMM controls (n=36-118 experiments in 6 PiZZ and 19 PiMM control animals; *P*-value by mixed effects model, *p*=0.136 for females and *p*=0.429 for males). (**C**) Elastance of the respiratory system (cmH_2O/ml) in PiZZ and PiMM control animals (n=36-118 experiments in 6 PiZZ and 19 PiMM control animals; *P*-value by mixed effects model, *p*=0.0443 in females and *p*=0.404 in males). (**D**) Quasi-static compliance (ml/cmH_2O) in PiZZ and PiMM control animals (n=36-118 experiments in 6 PiZZ and 19 PiMM control animals; *P*-value by mixed effects model, *p*=0.43 in females and *p*=0.111 in males). (**E**) $FEV_{0.4}$:FVC ratio over age in PiZZ ferrets. In all panels, squares indicate male ferrets and lighter colored circles indicate female ferrets. Panels A–D show mean±SEM and the number in parentheses indicate number of ferrets. Data are compared as indicated within the description for each graph. *$p<0.05$

Supplemental Figure 9

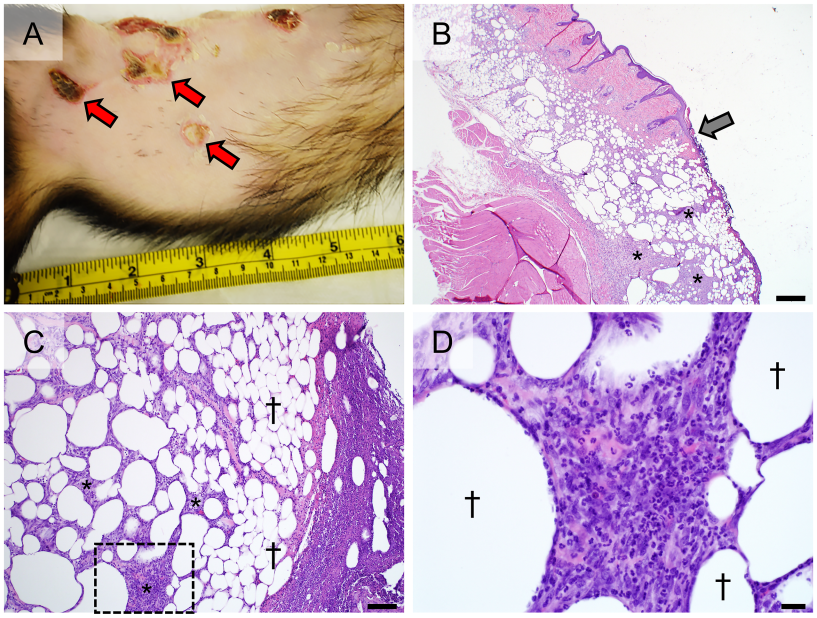

Supplemental Figure 9. Panniculitis in AAT-KO ferrets. (**A**) Deep ulcerative lesions on the trunk of affected AAT-KO ferret with raised, erythematous margins and alopecia, which was also observed in many affected ferrets. (**B**) Hematoxylin and Eosin (H&E) stained section through the border of one lesion where there is segmental epidermal ulceration and subjacent dermal inflammation. The grey arrow indicates the border of the lesion. Asterisks mark dermal and subcuticular inflammatory cell infiltrates. Scale bar represents 500µm. (**C**) Inflammatory cell infiltrates (asterisks) between areas of normal subcutaneous fat (daggers). Scale bar represents 100µm. (**D**) Detail of box in (C) showing that the inflammatory cell infiltrate is primarily neutrophils in the space between adipocytes (daggers). Scale bar represents 20µm.

References:

1. Li Z, Sun X, Chen J, Liu X, Wisely SM, Zhou Q, Renard JP, Leno GH, Engelhardt JF. Cloned ferrets produced by somatic cell nuclear transfer. *Dev Biol* 2006; 293: 439-448.
2. Yu M, Sun X, Tyler SR, Liang B, Swatek AM, Lynch TJ, He N, Yuan F, Feng Z, Rotti PG, Choi SH, Shahin W, Liu X, Yan Z, Engelhardt JF. Highly Efficient Transgenesis in Ferrets Using CRISPR/Cas9-Mediated Homology-Independent Insertion at the ROSA26 Locus. *Sci Rep* 2019; 9: 1971.
3. Rosen BH, Evans TIA, Moll SR, Gray JS, Liang B, Sun X, Zhang Y, Jensen-Cody CW, Swatek AM, Zhou W, He N, Rotti PG, Tyler SR, Keiser NW, Anderson PJ, Brooks L, Li Y, Pope RM, Rajput M, Hoffman EA, Wang K, Harris JK, Parekh KR, Gibson-Corley KN, Engelhardt JF. Infection Is Not Required for Mucoinflammatory Lung Disease in CFTR-Knockout Ferrets. *Am J Respir Crit Care Med* 2018; 197: 1308-1318.
4. Sun X, Yi Y, Yan Z, Rosen BH, Liang B, Winter MC, Evans TIA, Rotti PG, Yang Y, Gray JS, Park SY, Zhou W, Zhang Y, Moll SR, Woody L, Tran D, Jiang L, Vonk AM, Beekman JM, Negulescu P, Van Goor F, Fiorino D, Gibson-Corley KN, Engelhardt JF. In utero and postnatal VX-770 administration rescues multiorgan disease in a ferret model of cystic fibrosis. *Sci Transl Med* 2019; 11: 1-12.
5. Meyer KC, Raghu G, Baughman RP, Brown KK, Costabel U, du Bois RM, Drent M, Haslam PL, Kim DS, Nagai S. An official American Thoracic Society clinical practice guideline: the clinical utility of bronchoalveolar lavage cellular analysis in interstitial lung disease. *Am J Respir Crit Care Med* 2012; 185: 1004-1014.
