# Supplementary material for: Ferret models of alpha-1 antitrypsin deficiency develop lung and liver disease
Source: JCI Insight. 2022 Mar 8;7(5):e143004. doi: 10.1172/jci.insight.143004 (PMC8983124; doi:10.1172/jci.insight.143004)
Supplement: Supplemental table 1 [file jciinsight-7-143004-s037.pdf]

Supplemental Table 1. Genotypes of all F0 founders determined by Sanger sequencing. Indels are denoted by red letter bases for insertions and dashed for deletions.

```
Wt    ---TTGCAGGACGATGGCATCCTCCGTTCCCTGGGGCCTCCTGC---
```

No. T3 (ear tag: 164 MC# 047373820**) Female (DOB 1/23/16) (Chimera)**

```
Clone1---TTGCAGGACGATGGCATCCTC-----CCTGGGGCCTCCTGC---    -5 bp
Clone2---TTGCAGGACGATGGCATCCTC-----CCTGGGGCCTCCTGC---    -5 bp
Clone3---TTGCAGGACGATGGCATCCT-------------------GC---   -19 bp
Clone4---TTGCAGGACGATGGCATCCT-------------------GC---   -19 bp
Clone5---TTGCAGGACGATGGCATCCT-------------------GC---   -19 bp
Clone6---TTGCAGGACGATGGCATCCTCCGTTCCCTGGGGCCTCCTGC---    WT
```

No. S1 (ear tag: 117 MC# 047278792**) Male (DOB 1/24/16) (Chimera)**

```
Clone1---TTGCAGGACGA-----------------TGGGGCCTCCTGC---    -17 bp
Clone2---TTGCAGGACGATGGCATCCTCCGTTCCCCTGGGGCCTCCTGC---   +1  bp
Clone3---TTGCAGGACGA-----------------TGGGGCCTCCTGC---    -17 bp
Clone4---TTGCAGGACGATGGCATCCTCCGTTCCCTGGGGCCTCCTGC---    WT
Clone5---TTGCAGGACGA-----------------TGGGGCCTCCTGC---    -17 bp
Clone6---TTGCAGGACGATGGCATCCTCCGTTCCCCTGGGGCCTCCTGC---   +1  bp
```

No. F2 (ear tag: 423 MC# 841561775**) Female (DOB 3/22/16) (Homozygote)**

```
Clone1---TTGCAG---------------------------CCTCCTGC---     -27 bp
Clone2---TTGCAG---------------------------CCTCCTGC---     -27 bp
Clone3---TTGCAG---------------------------CCTCCTGC---     -27 bp
Clone4---TTGCAGGACGATGGCATCC--------CTGGGGCCTCCTGC---     -8  bp
Clone5---TTGCAGGACGATGGCATCC--------CTGGGGCCTCCTGC---     -8  bp
Clone6---TTGCAGGACGATGGCATCC--------CTGGGGCCTCCTGC---     -8  bp
```

No. S3 (ear tag: 174 MC# 047378330**) Male (DOB 1/24/16) (Heterozygote)**

```
Clone1---TTGCAGGACGATGGCATCCTCCGTTCCCTGGGGCCTCCTGC---    WT
Clone2---TTGCAGGACGATGGCATCCTCCGTTCCCTGGGGCCTCCTGC---    WT
Clone3---TTGCAGGACGATGG-----------------GGCCTCCTGC---    -17 bp
Clone4---TTGCAGGACGATGG-----------------GGCCTCCTGC---    -17 bp
Clone5---TTGCAGGACGATGG-----------------GGCCTCCTGC---    -17 bp
Clone6---TTGCAGGACGATGG-----------------GGCCTCCTGC---    -17 bp
```

No. S2 (ear tag: 297 MC# 047576529**) Male (DOB 1/24/16) (Heterozygote)**

```
Clone1---TTGCAGGACGATGGCATCCTCC-----CTGGGGCCTCCTGC---    -5 bp
Clone2---TTGCAGGACGATGGCATCCTCC-----CTGGGGCCTCCTGC---    -5 bp
Clone3---TTGCAGGACGATGGCATCCTCC-----CTGGGGCCTCCTGC---    -5 bp
Clone4---TTGCAGGACGATGGCATCCTCCGTTCCCTGGGGCCTCCTGC---    WT
Clone5---TTGCAGGACGATGGCATCCTCC-----CTGGGGCCTCCTGC---    -5 bp
Clone6---TTGCAGGACGATGGCATCCTCC-----CTGGGGCCTCCTGC---    -5 bp
```

No. S6 (ear tag: 342 MC# 047380027**) Male (DOB 1/24/16) (Heterozygote)**

```
Clone1---TTGCAGGACGATGGCATCCTCC-----CTGGGGCCTCCTGC---    -5 bp
Clone2---TTGCAGGACGATGGCATCCTCC-----CTGGGGCCTCCTGC---    -5 bp
Clone3---TTGCAGGACGATGGCATCCTCC-----CTGGGGCCTCCTGC---    -5 bp
Clone4---TTGCAGGACGATGGCATCCTCC-----CTGGGGCCTCCTGC---    -5 bp
Clone5---TTGCAGGACGATGGCATCCTCC-----CTGGGGCCTCCTGC---    -5 bp
Clone6---TTGCAGGACGATGGCATCCTCCGTTCCCTGGGGCCTCCTGC---    WT
```

No. U1 (ear tag: 251 MC# 047335111**) Male (DOB 1/30/16) (Heterozygote)**

```
Clone1---TTGCAGGACGATGGCATCC-----TCCCTGGGGCCTCCTGC---    -5 bp
Clone2---TTGCAGGACGATGGCATCC-----TCCCTGGGGCCTCCTGC---    -5 bp
Clone3---TTGCAGGACGATGGCATCC-----TCCCTGGGGCCTCCTGC---    -5 bp
```

```
Clone4---TTGCAGGACGATGGCATCCTCCGTTCCCTGGGGCCTCCTGC---    WT
Clone5---TTGCAGGACGATGGCATCC-----TCCCTGGGGCCTCCTGC---    -5 bp
Clone6---TTGCAGGACGATGGCATCCTCCGTTCCCTGGGGCCTCCTGC---    WT
```

No. S3 (ear tag: 199 MC# 047327842) **Female (DOB 1/24/16) (Heterozygote)**

```
Clone1---TTGCAGGACGATGGCATCCT--------TGGGGCCTCCTGC---     -8 bp
Clone2---TTGCAGGACGATGGCATCCT--------TGGGGCCTCCTGC---     -8 bp
Clone3---TTGCAGGACGATGGCATCCT--------TGGGGCCTCCTGC---     -8 bp
Clone4---TTGCAGGACGATGGCATCCTCCGTTCCCTGGGGCCTCCTGC---     WT
Clone5---TTGCAGGACGATGGCATCCTCCGTTCCCTGGGGCCTCCTGC---     WT
Clone6---TTGCAGGACGATGGCATCCTCCGTTCCCTGGGGCCTCCTGC---     WT
```

No. T5 (ear tag: 476 MC# 047548594) **Female (DOB 1/23/16) (Chimera)**

```
Clone1---TTGCAGGACGAT-------------------GGCCTCCTGC---     -19 bp
Clone2---TTGCAGGACGAT-------------------GGCCTCCTGC---     -19 bp
Clone3---TTGCAGGACGATGGCATCCTCC-----CTGGGGCCTCCTGC---     -5  bp
Clone4---TTGCAGGACGATGGCATCCTCCGTTCCCTGGGGCCTCCTGC---     WT
Clone5---TTGCAGGACGATGGCATCCTCCGTTCCCTGGGGCCTCCTGC---     WT
Clone6---TTGCAGGACGATGGCATCCTCCGTTCCCTGGGGCCTCCTGC---     WT
```

NO. C4 (ear tag: 369) Male (DOB 3/11/16) (Chimera)

```
Clone1---TTGCAGGACGATGGCATCCTCCGTTCCCTGGGGCCTCCTGC---     WT
Clone2---TTGCAGGACGATGGCATCCTCCGTTCCCTGGGGCCTCCTGC---     WT
Clone3---TTGCAGGACGATGGCATCCTCCGT-----GGGGCCTCCTGC---     -5 bp
Clone4---TTGCAGGACGATGGCATCCTCCGT-----GGGGCCTCCTGC---     -5 bp
Clone5---TTGCAGGACGATGGCATCCTCCGT-----GGGGCCTCCTGC---     -5 bp
Clone6---TTGCAGGACGATGGCATCCTCCGT-CCCTGGGGCCTCCTGC---     -1 bp
```

NO. C2 (ear tag: 252) Female (DOB 3/11/16) (Chimera)

```
Clone1---TTGCAGGACGATGGCATCCTCCGTTCCCTGGGGCCTCCTGC---     WT
Clone2---TTGCAGGACGATGGCATCCTCCGTTCCCTGGGGCCTCCTGC---     WT
Clone3---TTGCAGGACGATGGCATCC--------CTGGGGCCTCCTGC---     -8 bp
Clone4---TTGCAGGACGATGGCATCC--------CTGGGGCCTCCTGC---     -8 bp
Clone5---TTGCAGGACGATGGCATCC--------CTGGGGCCTCCTGC---     -8 bp
Clone6---TTGCAGGACGATGGCATCCTCCGTCCCCTGGGGCCTCCTGC---     +1 bp
```

NO. B2 (ear tag: 368) Female (DOB 3/13/16) (Homozygote)

```
Clone1---TTGCAGGACGATGGCATCCTC----CCCTGGGGCCTCCTGC---     -4 bp
Clone2---TTGCAGGACGATGGCATCCTCCGTTCCCCTGGGGCCTCCTGC---    +1 bp
Clone3---TTGCAGGACGATGGCATCCTCC----CCTGGGGCCTCCTGC---     -4 bp
Clone4---TTGCAGGACGATGGCATCCTCC----CCTGGGGCCTCCTGC---     -4 bp
Clone5---TTGCAGGACGATGGCATCCTCC----CCTGGGGCCTCCTGC---     -4 bp
Clone6---TTGCAGGACGATGGCATCCTCC----CCTGGGGCCTCCTGC---     -4 bp
```

No. E3 (ear tag: 370) Male (DOB 3/17/16) (Homozygote)

```
Clone1---TTGCAGGACGATGGCATCCTCC-----CCTGGGGCCTCCTGC---    -5 bp
Clone2---TTGCAGGACGATGGCATCCTCC-----CCTGGGGCCTCCTGC---    -5 bp
Clone3--- a large deletion
Clone4---TTGCAGGACGATGGCATCCTCCGT---CCTGGGGCCTCCTGC---    -3 bp
Clone5---TTGCAGGACGATGGCATCCTCC-----CCTGGGGCCTCCTGC---    -5 bp
Clone6---TTGCAGGACGATGGCATCCTCC-----CCTGGGGCCTCCTGC---    -5 bp
```
