# Supplementary material for: Ferret models of alpha-1 antitrypsin deficiency develop lung and liver disease
Source: JCI Insight. 2022 Mar 8;7(5):e143004. doi: 10.1172/jci.insight.143004 (PMC8983124; doi:10.1172/jci.insight.143004)
Supplement: Supplemental table 2 [file jciinsight-7-143004-s041.pdf]

Supplemental Table 2. Genetic background and age at the time of flexiVent PFT for AAT-KO ferrets used in the study.

Micro Chip	DOB	Gender (M/F)	Generation (F#)	Breeding pair Hobb (M)	Breeding pair Jill (F)	Genotype (Indel/insert)	flexiVent, PFTs (age in days)
#117	1/24/16	M	F0	WT	WT	-17/+1	540, 570, 597, 639, 673, 718, 822, 858
#423	3/22/16	F	F0	WT	WT	-27/-8	485, 512, 540, 581, 615, 645
838768813	10/24/16	F	F1	#117 F0	#164 F0	-17/-5	273, 301, 325, 366, 399, 583, 676, 710, 738, 773, 849, 870
838639798	10/24/16	F	F1	#117 F0	#164 F0	-19/+1	267, 301, 325, 365, 399, 540, 582, 676, 714, 739, 773, 849, 870
843520987	2/27/17	F	F1	#117 F0	#423 F0	-27/+1	158, 221, 240, 281, 339, 381, 401, 444, 548, 585
843514359	2/27/17	F	F1	#117 F0	#423 F0	-27/-17	205, 241, 281, 309, 346, 382, 402, 443, 498, 549
843515865	2/27/17	F	F1	#117 F0	#423 F0	-27/+1	205, 241, 281, 315, 338, 399, 456, 550, 585, 612, 647, 687, 744, 1032
843514181	2/27/17	M	F1	#117 F0	#423 F0	-27/+1	205, 240, 281, 318, 423, 458, 548, 588, 611, 647, 729, 744
842803550	9/19/17	M	F2	#838768560 (-17/WT)	#843521331 (+1/WT)	-17/+1	344, 379, 406, 442, 524, 540
#797	11/8/18	M	F2	#843514823	#842807122	-17/-17	449
#559	11/14/18	M	F2	#842788567	#838608524	-17/-17	343
#274	11/14/18	M	F2	#842788567	#838608524	-17/-17	443
#586	11/14/18	M	F2	#842788567	#838608524	-17/-17	443
AAT-KO ferrets used for LPS injury experiment:							
#377	6/22/18	M	F2	#842803550	#843515865	-27/+1	207
#620	7/4/18	M	F2	#843514823	#842807035	-17/-5	222
#846	8/30/18	F	F2	#838609109	#842807122	-17/-17	169
#833	8/30/18	F	F2	#838609109	#842807122	-17/-17	169
#788	7/4/18	M	F2	#843514823	#842807035	-17/-5	196
#084	8/30/18	M	F2	#838609109	#842807122	-17/-17	140
#562	7/4/18	F	F2	#843514823	#842807035	-17/-5	196
#855	7/4/18	F	F2	#838609109	#842797061	-17/-17	196

Check Suppl. Table 1 for founders' (F0) information (ID # and genotyping).
Abbreviations: F, female; M, male; WT, wild type.
