# Supplementary material for: Ferret models of alpha-1 antitrypsin deficiency develop lung and liver disease
Source: JCI Insight. 2022 Mar 8;7(5):e143004. doi: 10.1172/jci.insight.143004 (PMC8983124; doi:10.1172/jci.insight.143004)
Supplement: Supplemental table 3 [file jciinsight-7-143004-s042.pdf]

Supplemental Table 3. Ages of control ferrets used to control for AAT-KO flexiVent PFT experiments.

Micro Chip	DOB	Gender (M/F)	Generation (F#)	Breeding pair		Genotype (Indel/insert)	flexiVent, PFTs (age in days)
				Hobb (M)	Jill (F)		
Ctrl 117	1/24/16	M	WT	WT	WT	WT/WT	541, 576, 597, 633, 676, 710, 814, 850
Ctrl 423	3/22/16	F	WT	WT	WT	WT/WT	485, 512, 556, 603, 637, 675
Ctrl 813	10/24/16	F	WT	WT	WT	WT/WT	273, 301, 693, 717, 752, 828, 849
Ctrl 798	10/24/16	F	WT	WT	WT	WT/WT	267, 301, 325, 367, 399, 544, 608, 680, 718, 743, 777, 852, 874
Ctrl 987	2/27/17	F	WT	WT	WT	WT/WT	156, 219, 344, 399, 442, 547, 584
Ctrl 359	2/27/17	F	WT	WT	WT	WT/WT	185, 220, 260, 287, 360, 381, 442, 529, 564
Ctrl 865	2/27/17	F	WT	WT	WT	WT/WT	185, 220, 288, 322, 378, 435, 528, 565, 592, 626, 666, 722, 1011
Ctrl 181	2/27/17	M	WT	WT	WT	WT/WT	226, 250, 292, 326, 431, 466, 557, 596, 619, 655, 737, 753
Ctrl 550	9/19/17	M	WT	WT	WT	WT/WT	378, 406, 442, 524, 540
Ctrl 797	11/8/18	M	WT	WT	WT	WT/WT	472
Ctrl 559	11/14/18	M	WT	WT	WT	WT/WT	374
Ctrl 274	11/14/18	M	WT	WT	WT	WT/WT	517
Ctrl 586	11/14/18	M	WT	WT	WT	WT/WT	445
Control ferrets used for LPS injury experiment:							
Ctrl 377	7/4/18	M	WT	WT	WT	WT/WT	195
Ctrl 620	7/4/18	M	WT	WT	WT	WT/WT	197
Ctrl 846	8/30/18	F	WT	WT	WT	WT/WT	169
Ctrl 833	8/30/18	F	WT	WT	WT	WT/WT	169
Ctrl 788	6/22/18	M	WT	WT	WT	WT/WT	209
Ctrl 084	7/4/18	M	WT	WT	WT	WT/WT	196
Ctrl 562	7/4/18	F	WT	WT	WT	WT/WT	196
Ctrl 855	8/30/18	F	WT	WT	WT	WT/WT	177

Abbreviations: F, female; M, male; WT, wild type.
