# Supplementary material for: Ferret models of alpha-1 antitrypsin deficiency develop lung and liver disease
Source: JCI Insight. 2022 Mar 8;7(5):e143004. doi: 10.1172/jci.insight.143004 (PMC8983124; doi:10.1172/jci.insight.143004)
Supplement: Supplemental table 4 [file jciinsight-7-143004-s043.pdf]

Supplemental Table 4. Age at which CT scans and BAL were obtained from AAT-KO and matched controls, with control ages in parentheses.

Micro Chip	DOB	Gender (M/F)	Generation (F#)	Breeding pair		Genotype (Indel/insert)	CT scan: KO (Ctrl), age in days	BAL: KO (Ctrl), age in days
				Hobb (M)	Jill (F)			
#117	1/24/16	M	F0	WT	WT	-17/+1	850 (919)	535 (536)
#423	3/22/16	F	F0	WT	WT	-27/-8	n/a	422 (421)
838768813	10/24/16	F	F1	#117 F0	#164 F0	-17/-5	682 (714)	n/a
838639798	10/24/16	F	F1	#117 F0	#164 F0	-19/+1	735 (739)	392 (388)
843520987	2/27/17	F	F1	#117 F0	#423 F0	-27/+1	416 (414)	401 (399)
843514359	2/27/17	F	F1	#117 F0	#423 F0	-27/-17	556 (536)	402 (381)
843515865	2/27/17	F	F1	#117 F0	#423 F0	-27/+1	1040 (1019)	399 (388)
843514181	2/27/17	M	F1	#117 F0	#423 F0	-27/+1	557 (565)	325 (333)
842803550	9/19/17	M	F2	#838768560	#843521331	-17/+1	352 (385)	n/a

Abbreviations: F, female; M, male; WT, wild type; n/a, not available.
