# Supplementary material for: Ferret models of alpha-1 antitrypsin deficiency develop lung and liver disease
Source: JCI Insight. 2022 Mar 8;7(5):e143004. doi: 10.1172/jci.insight.143004 (PMC8983124; doi:10.1172/jci.insight.143004)
Supplement: Supplemental table 5 [file jciinsight-7-143004-s044.pdf]

Supplemental Table 5. Current status of animals in the AAT-KO study or age at which ferret was euthanized

Micro Chip	DOB	Gender (M/F)	Generation (F#)	Breeding pair		Genotype (Indel/insert)	Alive or euthanized (age in days)
				Hobb (M)	Jill (F)		
#117	1/24/16	M	F0	WT	WT	-17/+1	Euthanized, 879d (end-point)
#423	3/22/16	F	F0	WT	WT	-27/-8	Euthanized, 650d (end-point)
838768813	10/24/16	F	F1	#117 F0	#164 F0	-17/-5	Alive, >1296d
838639798	10/24/16	F	F1	#117 F0	#164 F0	-19/+1	Euthanized, 921d (end-point)
843520987	2/27/17	F	F1	#117 F0	#423 F0	-27/+1	Euthanized, 581d
843514359	2/27/17	F	F1	#117 F0	#423 F0	-27/-17	Euthanized, 581d (panniculitis)
843515865	2/27/17	F	F1	#117 F0	#423 F0	-27/+1	Alive, >1170d
843514181	2/27/17	M	F1	#117 F0	#423 F0	-27/+1	Alive, >1170d
842803550	9/19/17	M	F2	#838768560	#843521331	-17/+1	Euthanized, 562d (end-point)
#797	11/8/18	M	F2	#843514823	#842807122	-17/-17	Alive, >551d
#559	11/14/18	M	F2	#842788567	#838608524	-17/-17	Euthanized 382d (failure to thrive)
#274	11/14/18	M	F2	#842788567	#838608524	-17/-17	Alive, >545d
#586	11/14/18	M	F2	#842788567	#838608524	-17/-17	Alive, >545d
AAT-KO ferrets used for LPS injury experiment:							
#377	6/22/18	M	F2	#842803550	#843515865	-27/+1	Euthanized, 256d (end-point)
#620	7/4/18	M	F2	#843514823	#842807035	-17/-5	Euthanized, 247d (end-point)
#846	8/30/18	F	F2	#838609109	#842807122	-17/-17	Euthanized, 223d (end-point)
#833	8/30/18	F	F2	#838609109	#842807122	-17/-17	Euthanized, 224d (end-point)
#788	7/4/18	M	F2	#843514823	#842807035	-17/-5	Euthanized, 257d (end-point)
#084	8/30/18	M	F2	#838609109	#842807122	-17/-17	Euthanized, 203d (end-point)
#562	7/4/18	F	F2	#843514823	#842807035	-17/-5	Euthanized, 264d (end-point)
#855	7/4/18	F	F2	#838609109	#842797061	-17/-17	Euthanized, 267d (end-point)

Abbreviations: F, female; M, male; WT, wild type.
