# Supplementary material for: Ferret models of alpha-1 antitrypsin deficiency develop lung and liver disease
Source: JCI Insight. 2022 Mar 8;7(5):e143004. doi: 10.1172/jci.insight.143004 (PMC8983124; doi:10.1172/jci.insight.143004)
Supplement: Supplemental table 10 [file jciinsight-7-143004-s038.pdf]

Supplemental Table 10. Genetic background and flexiVent PFT ages of PiZZ ferrets used for characterization

Micro Chip	DOB	Gender (M/F)	Generation (F#)	Breeding pair		Genotype (Indel/insert)	flexiVent, PFTs (age in days)
				Hobb (M)	Jill (F)		
#344	11/15/17	F	F1	#838617559	#842797319	Z/Z	121, 153, 181, 212, 239, 267, 328, 357, 386, 461, 482
#334	11/15/17	M	F1	#838617559	#842797319	Z/Z	121, 152, 181, 212, 292, 386, 812
#776	2/5/19	F	F2	#842798809	#842788771	Z/Z	135, 213, 240, 366, 528
#518	5/29/19	F	F2	#842798809	#841560786	Z/Z	100, 141, 188, 252, 465
#046	6/4/19	M	F2	#845093065	#842788771	Z/Z	94, 135, 182, 246, 409, 435
#881	7/24/19	M	F2	#845263777	#845263591	Z/Z	197, 366

Abbreviations: F, female; M, male; WT, wild type.
