# Supplementary material for: Ferret models of alpha-1 antitrypsin deficiency develop lung and liver disease
Source: JCI Insight. 2022 Mar 8;7(5):e143004. doi: 10.1172/jci.insight.143004 (PMC8983124; doi:10.1172/jci.insight.143004)
Supplement: Supplemental table 11 [file jciinsight-7-143004-s039.pdf]

Supplemental Table 11. Genetic background and flexiVent PFT ages of ferrets used to control for PiZZ experiments.

Micro Chip	DOB	Gender (M/F)	Generation (F#)	Breeding pair		Genotype (Indel/insert)	flexiVent, PFTs (age in days)
				Hobb (M)	Jill (F)		
Ctrl 344	11/15/17	F	WT	WT	WT	M/M	122, 151, 185, 213, 339, 371, 384, 448, 478
Ctrl 334	11/15/17	M	WT	WT	WT	M/M	161, 212, 292
Ctrl 776	2/5/19	F	WT	WT	WT	M/M	135, 213, 240, 381, 532
Ctrl 518	5/29/19	F	WT	WT	WT	M/M	135, 182, 261, 449
Ctrl 046	6/4/19	M	WT	WT	WT	M/M	136, 182, 414, 436
Ctrl 881	7/24/19	M	WT	WT	WT	M/M	204, 343

Abbreviations: F, female; M, male; WT, wild type.
