# Supplementary material for: Ferret models of alpha-1 antitrypsin deficiency develop lung and liver disease
Source: JCI Insight. 2022 Mar 8;7(5):e143004. doi: 10.1172/jci.insight.143004 (PMC8983124; doi:10.1172/jci.insight.143004)
Supplement: Supplemental table 12 [file jciinsight-7-143004-s040.pdf]

Supplemental Table 12. Primer and probes for genotyping and quantitative PCR.

Primer/probe set	Forward/Reverse	Sequence (5'→3')	Amplicon (bp)
AAT-KO genotyping	Forward	TGTGCCCTAGGTCATAAGTC	942
	Reverse	TGGTGTTGGCAACCTTAC	
AAT-PiZZ genotyping	Forward	GAAGGAGCCACGGAAGATGAATG	1069
	Reverse	ATGGAAAGCGGACCGAGTAG	
GAPDH qPCR	Forward	CAACTTTGGCATTGTGGAGG	NA
	Reverse	CAGTGGAAGCAGGGATGATG	
	Probe	CAGTGATGGCATGGACGGTGG	
AAT qPCR	Forward	TTCTTTAAAGGCAAATGGGAGAAG	NA
	Reverse	ATCATGGGCACCTTGACG	
	Probe	AGCACACCACAGTGGAGGACTTC	
